# GC–MS-based metabolome classification of sturgeon caviar and fish roe samples reveals unique caviar signatures, interspecies and gender variabilities

**DOI:** 10.1038/s41598-026-36474-6

**Published:** 2026-02-17

**Authors:** Nehal Ibrahim, Amira R. Khattab, Ashraf S. Mohammad, Montasser A. Al-Hammady, Iriny M. Ayoub, Mohamed A. Farag

**Affiliations:** 1 Pharmacognosy Department, Faculty of Pharmacy, Ain Shams University, Cairo, 11566 Egypt; 2 Pharmacognosy Department, College of Pharmacy, Arab Academy for Science, Technology and Maritime Transport, Alexandria, 1029 Egypt; 3 Graduate School in Alamein, Arab Academy for Science, Technology & Maritime Transport, Alamein, Egypt; 4 National Institute of Oceanography and Fisheries (NIOF), Cairo, 11516 Egypt; 5 Pharmacognosy Department, College of Pharmacy, Cairo University, Kasr El Aini St., P.B. 11562, Cairo, Egypt

**Keywords:** Caviar, Fish roe, GC–MS, Metabolomics, OPLS-DA, Chemometrics, Biochemistry, Zoology

## Abstract

Caviar/roe, widely valued in modern cuisine, is little characterized regarding its complete metabolite composition. Existing studies focused primarily on specific classes e.g., lipids and proteins. This study represents the first extensive GC–MS metabolite profiling of 48 caviar/roe samples from 10 commercially important taxa, including male and female aquatic animals. One hundred thirty-nine primary and secondary metabolites were identified and quantified belonging to fatty, amino, and organic acids, sugars, nitrogenous compounds, and steroids/terpenoids. Multivariate data analysis further uncovered clear interspecies and gender-specific metabolic differences. OPLS-DA highlighted palmitic acid and cholesterol as discriminative markers for sturgeon caviar, while serine and urea distinguished salmon roe. Gender differences were evident in *Charybdis natator* roe, with males enriched in amino acids and females in fatty metabolites. This comprehensive caviar/roe metabolite map proposes male gilt-head bream *Sparus aurata* and female common cuttlefish *Sepia officinalis* for further investigation of their potential functional food applications, driven by their rich omega-3 PUFA levels. Specifically, *S. officinalis* roe presents good fat source being rich in γ-tocopherol, with favorable n-3/n-6 ratio. The rich content of pyroglutamic acid in *S. aurata* may contribute to its characteristic umami taste encouraging further sensory analysis. The findings strengthen the molecular basis for improved quality assessment and nutritional labeling of caviar/roe products.

**Supplementary Information:**

The online version contains supplementary material available at 10.1038/s41598-026-36474-6.

## Introduction

Caviar is one of the finest seafood delicacies due to its sensory, socio-cultural, and nutritional properties. Genuine black caviar is the salt-cured roe of over 20 sturgeon fish species e.g., *Huso huso*, *Acipenser gueldenstaedtii*, *A. stellatus*, and *A. baeri* found primarily in the Black and Caspian seas. In contrast, inferior types include acipenseriformes paddlefish species *Polyodon spathula*^1,2^. The overexploitation of sturgeons for caviar production led to a dramatic decline in the wild sturgeon population, with almost all sturgeon species currently critically endangered^3^. Consequently, caviar supply was shifted from wild to farmed sturgeons to support the supply chain. Furthermore, roe from non-sturgeon species is widely used as a less costly caviar substitute and is preferentially named roe rather than caviar^1^.

A wide range of roe can be obtained from non-sturgeon species such as salmon roe referred to as red caviar which is the most popular caviar substitute, as well as trout, cod, and mullet roes^2^. Other aquatic animals are regarded as less common sources of roe including cephalopods as squid and cuttlefish, echinoderms as sea urchins, and crustaceans as shrimp and crab. Although black caviar is the most valued roe, the global consumption of roe from other aquatic species is higher considering the globalization of sushi culture. Of the 60,000 tons of fish roes produced annually, black caviar represents only 1%^4^.

Asides from its unique sensory traits triggered by its umami taste and desirable texture, caviar/roe is endowed with nutritive properties due to its high-quality protein rich in essential amino acids. Different amino acid profiles were reported in various roe types, with key amino acids as glycine, alanine, aspartic acid, and glutamic acid, with glutamic acid as a chief contributor to caviar/roe taste^5^. Despite the variable fatty acid profile in different roe types, there is generally a significant content of polyunsaturated fatty acids (PUFA), e.g. eicosapentaenoic and docosahexaenoic acids, which play a role in oxidative homeostasis and cardiometabolic health^1,6^. As a seafood, roe presents a favored source of n-3/n-6 PUFA ratio, which is a potential indicator of fat quality and can reach 20 in herring roe^7^. Meanwhile, several factors can affect the chemical makeup of roe, hence its bio-functional value and sensory characteristics, such as fish species, habitat, processing methods, and roe stage of maturity^1^.

Over the last decades, fish consumption increased worldwide at a significant rate that outpaced the population growth^8^. For many of the aquatic species consumed for their meat, roe is discarded or at best utilized as fish feed^9^. Considering that roe can reach up to 30% of the fish’s whole body mass, exploitation in the human diet capitalizes on fish resources, reduces waste and ecological burden, and identifies novel nutritious food^8^. Comprehensive biochemical profiling of roe is a prerequisite for prioritizing valuable roe sources of potential benefits or health hazards, if any.

The holistic mapping of food metabolome using large-scale analytical platforms represented by hyphenated mass spectrometry techniques, GC–MS or LC–MS, results in the generation of complex data matrices which are best harnessed by chemometric tools to reduce dimensionality, visualize differences, generate classification models, and discriminate between different groups^10,11^. Despite the potential impact of caviar/roe products on the modern international food market, few studies have described the variability of biochemical makeup among different types of fish roe employing single class analysis, namely lipids or proteins rather than the full metabolomic mapping in the analysis of fish roe^12,13^. Scarce reports have employed multivariate data analysis (MVA) mostly represented by principal component analysis (PCA) in fish roe classification, and relying on fatty acids class^14–16^, with less information on other nutrient classes potential in fish roe classification. A recent study implemented LC–MS with MVA tools to evaluate the impact of processing conditions on sturgeon caviar metabolome^17^. However, this study did not analyze non-sturgeon fish roe in comparison to sturgeon caviar.

The present study aimed to provide comprehensive GC–MS-based qualitative and quantitative metabolites profiling of caviar and roe from different marine organisms. Forty-eight roe samples were analyzed originating from different genotypes and genders belonging to 5 fishes, namely *Oncorhynchus keta*/*O. gorbuscha*, *Huso*/*Acipenser* species, *Rhabdosargus haffara*, *Sparus aurata,* and *Dicentrarchus labrax,* and five aquatic animals; *Charybdis natator*, *Portunus pelagicus*, *Tripneustes gratilla*, *Sepia officinalis* and *Sepioteuthis lessoniana* (Table 1). The study was designed to cover roe from various marine organisms of economic importance (food fish caught in large quantities) in comparison with genuine black caviar (BCV) and salmon roe (RCV). Family Sparidae (porgies) which holds notable commercial importance is represented here by one haffara seabream (*R. haffara*) roe sample originating from the Red Sea (RHF) and 4 gilt-head bream (*S. aurata*) roe samples obtained from the Mediterranean Sea and reflecting both male (SAM-1, SAM-2) and female fish (SAF-1, SAF-2). In addition, one European seabass (*D. labrax*) roe sample (DLF) was included to represent the temperate basses (Moronidae). European seabass is the most important commercial fish extensively cultured in the Mediterranean region. Crustaceans’ roe is exemplified by crab roe of family Portunidae including male and female ridged swimming crab; *C. natator* from the Red Sea (CNM, CNF) and the commercially popular blue swimming crab; *P. pelagicus* from the Mediterranean Sea (PPF-1, PPF-2). Among cephalopods, the common cuttlefish; *S. officinalis* and the bigfin reef squid; *S. lessoniana* are popular food items and a target of large-scale Mediterranean fisheries. Gender diversity in cephalopods roe was reflected by male and female *S. officinalis* specimens (SOM, SOF) and *S. lessoniana* roe (SLB). A roe sample from the collector urchin *T. gratilla* (TGF) represents echinoderms (Table 1). To provide insight into caviar/roe metabolome heterogeneity in the context of interspecies, gender-related, and infraspecific variability, different models of unsupervised and supervised chemometric tools were employed, exemplified by principal component analysis (PCA), hierarchical cluster analysis (HCA), and orthogonal partial least squares discriminant analysis (OPLS-DA). The implementation of GC–MS-based metabolomics to classify roe accessions in context to taxa and gender type originating from the Mediterranean and Red Sea, in comparison to black sturgeon caviar, is presented for the first time.

**Table 1:** Roe samples for GC–MS-based metabolome analysis.

Sample code	Name	Family	Sex	Source
RCV	*Oncorhynchus keta*/*O. gorbuscha* (Salmon)	Salmonidae	Female	Commercial
BCV	*Huso*/*Acipenser* sp (Sturgeon)	Acipenseridae	Female	Commercial
RHF	*Rhabdosargus haffara* (Haffara seabream)	Sparidae (Porgies)	Female	Red Sea
SAM-1	*Sparus aurata* (Gilt-head bream)	Sparidae (Porgies)	Male	Mediterranean Sea
SAF-1			Female	
SAM-2			Male	
SAF-2			Female	
CNM	*Charybdis natator* (Ridged swimming crab)	Portunidae	Male	Red Sea
CNF			Female	
PPF-1	*Portunus pelagicus* (Blue swimming crab)		Female	Mediterranean Sea
PPF-2			Female	
TGF	*Tripneustes gratilla* (Collector urchin)	Toxopneustidae	Female	Red Sea
DLF	*Dicentrarchus labrax* (European seabass)	Moronidae (Temperate basses)	Female	Mediterranean Sea
SOF	*Sepia officinalis* (Common cuttlefish)	Sepiidae	Female	Mediterranean Sea
SOM			Male	
SLB	*Sepioteuthis lessoniana* (Bigfin reef squid)	Loliginidae	Male and Female	Red Sea

## Materials and methods

### Caviar and roe samples

Experiments were conducted under approval of the Ethics Committee for the use of animal subjects, Faculty of Pharmacy, Ain Shams University, Cairo, Egypt (approval number REC 413). Roe samples were collected from the fish landing sites in Alexandria, Mediterranean Sea; site 1 (Yacht and Boat Marina, Anfoshy, Alexandria, 31°12′37.41"N, 29°52′58.87"E) and Hurghada, Red Sea; site 2 (El-Saqala fishing area, Hurghada, 27°13′43.25"N, 33°50′33.16"E), Egypt. The collection sites are depicted in the Supplementary Fig. S1.

Two different fishing trawlers were used at the current study; one trawler was specific for fishing in the Red Sea and another one was approved for sailing and fishing in the Mediterranean Sea. These trawlers are owned by local fishermen, licensed by local authorities and are subject to inspection, health and occupational safety procedures. To ensure the application of fish handling ethics during fishing operations, whether in the Red Sea or the Mediterranean Sea, fishing methods were reviewed by the authors Ashraf S. Mohammad and Montasser A. Al-Hammady to ensure they were appropriate. The study was conducted in compliance with national guidelines (Law No.146 of 2021 for the Protection and Development of Lakes and Fisheries). Samples were caught at sunrise, the most suitable time for marine organisms to feed, from the designated fishing areas. After the capture, the organisms were left free on the vessel allowing them to calm down and acclimate to the outside environment. After using the anesthetic agent tricaine methanesulfonate (MS-222), in dose of 90–110 µg/mL^18^, they died in complete silence, as marine organisms are not handled or opened alive. The collected organisms were stored in a cool environment (refrigerator, ice). Sterile and suitable dissection tools were used to collect samples, and a researcher specialized in marine fisheries was used. Fish roes from different animals were lyophilized to complete dryness overnight and kept in air-tight glass vessels at −20 °C till further analysis.

### Chemicals

N-Methyl-N- (trimethylsilyl)trifluoroacetamide (MSTFA) and xylitol were purchased from Sigma Aldrich (St. Louis, MO, U.S.A.). Alkane standard (C8-C40) and all other standards were purchased from Sigma Aldrich (St. Louis, MO, U.S.A.)

### Sample preparation for GC–MS analysis

Primary metabolites were analyzed as described in^17,19^. Briefly, freeze-dried and finely powdered caviar and roe samples (100 mg) were extracted with 1 ml 100% methanol spiked with xylitol as an internal standard at 10 µg/ml with sonication for a 5 min period, then centrifuged at 12,000 rpm for 10 min to eliminate the debris. 100 µl were aliquoted from the methanol extract, evaporated to dryness under a stream of nitrogen gas till complete dryness. Derivatization was carried out using 150 μL of MSTFA at 60 °C for 45 min, followed by equilibration at 28 °C before GC–MS analysis.

### GC–MS analysis

Samples were analyzed in triplicate under the same conditions. A Shimadzu QP-2010 gas chromatograph coupled to a mass spectrometer fitted with an Rtx-5MS column (30 m length, 0.25 μm film thickness, and 0.25 mm internal diameter) was used. A split mode with a 1:10 split ratio was used for injections under the following conditions: injector temperature set to 280 °C, column oven temperature was held at 70 °C for 3 min, then increased to 315 °C at a rate of 10 °C/min, and kept at 315 °C for 6 min. Helium was used as a carrier gas with a flow rate 1.24 mL/min. Interface and ion source temperatures were set to 280 °C and 180 °C, respectively. Electron ionization mode (EI, 70 eV) was employed with a scan range of *m/z* 35–500. Validation of GC–MS method has been previously reported^20^. Representative raw GC–MS data files are available through this shared drive link: https://drive.google.com/drive/folders/1eYqcipVWeQcniET_gh9UjKZaz079BBDv?usp=drive_link

#### Metabolites identification and absolute quantification

For identification, peaks were initially deconvoluted using AMDIS software (www.amdis.net). Silylated metabolites were then tentatively identified by comparing their retention indices (RI) with those of an *n*-alkane series (C8–C40), and matching their mass spectra to NIST and Wiley library databases following the procedure previously described^21^. Only annotations showing a matching score above 800 are considered. Peak abundance was obtained using MS-DIAL software with previously described parameters^20^. Fatty acids, organic acids, free amino acids, alcohols, and soluble sugars were quantified using standard curves for stearic acid, lactic acid, glycine, glycerol, and glucose, expressed as mg/g. Standard curves were constructed using four serial dilutions (10–600 µg/mL). Calibration curves for stearic acid, glycine, and glucose displayed a correlation coefficient of approximately 0.994821 following the same protocol described in^20^.

#### Multivariate data analysis

Multivariate data analysis was performed using unsupervised principal component analysis (PCA) and hierarchical cluster analysis (HCA), as well as supervised orthogonal partial least squares-discriminant analysis (OPLS-DA) with SIMCA 14.1 (Umetrics, Umea, Sweden). All variables were scaled and mean-centered to Pareto Variance. Unsupervised PCA was conducted to provide a comprehensive overview of metabolite variance among the different caviar specimens, meanwhile, supervised OPLS-DA was applied to validate PCA results and to obtain detailed insights into differences in metabolites composition among the studied samples. Chemometric models were evaluated employing R^2^ and Q^2^ parameters, with the number of permutations set at 200. R^2^ assessed the model’s goodness of fit, while Q^2^ indicated its predictability. Outliers were identified using DModx (distance to the model), and strong outliers in the OPLS-DA plot were detected via Hotelling’s T^2^. An iterative permutation test was performed to eliminate the non-random separation among groups.

#### Statistical analysis

All data were expressed as mean ± S.D. from three biological replicates per group. Two-way analysis of variance (ANOVA) followed by Tukey’s Post-Hoc test was performed using GraphPad Prism 9 for multiple comparisons among marker metabolites and metabolite classes at a significance level less than 0.05.

### Ethics statement

The experimental design was approved by the Ethics Committee for the use of animal subjects, at Ain Shams University, Cairo, Egypt (approval number REC 413). All experiments were performed in accordance with relevant guidelines and regulations and were reported in compliance with ARRIVE guidelines.

## Results and discussion

### GC–MS metabolome identification in caviar and roe samples

**Fig. 1:**
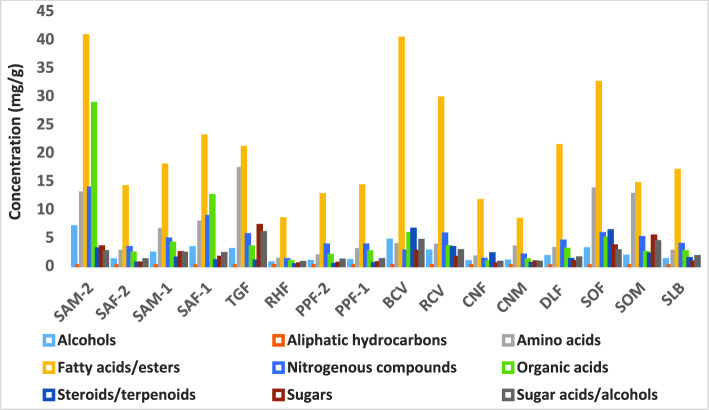
The concentration of major metabolite classes in caviar/roe samples expressed in mg/g. For samples codes, refer to Table 1.

GC–MS analysis was employed for profiling primary metabolites in all caviar and roe samples (see Table 1 for detailed samples’ information and codes) to include 139 peaks belonging to different classes i.e. fatty acids/esters (37 peaks), alcohols (12), aliphatic hydrocarbons (2), amino acids (27), nitrogenous compounds (14), organic acids (21), sugars (12), sugar alcohols/acids (9) as well as secondary metabolites exemplified by steroids/terpenoids (5) (Table 2). The level of major metabolite classes identified in caviar/roe specimens is illustrated in Fig. 1. GC–MS chromatograms of red caviar (salmon roe), genuine black caviar, as well as roe samples of the ridged swimming crab *Charybdis natator*, the collector urchin *Tripneustes gratilla*, the common cuttlefish *Sepia officinalis*, and the gilt-head bream *Sparus aurata* are depicted in Supplementary Fig. S2. GC–MS peak list is provided in Supplementary datasheet S1. The biological variance within each caviar or roe specimen was assessed by analyzing three independent biological replicates as detailed for each class in the next subsections.

**Table 2:** Concentration of silylated primary and secondary metabolites in caviar and roe samples analyzed via GC–MS, n = 3, and expressed as mean ± standard deviation (mg/g). For code explanation, refer to Table 1. Annotations followed by an asterisk indicate those confirmed with standards.

Peak no	Rt (min)	RI	Annotation^#^	SAM-2	SAF-2	SAM-1	SAF-1	TGF	RHF	PPF-2	PPF-1
2	5.37	989	Ethylene glycol 2TMS	0.31 ± 0.06	0.25 ± 0.02	0.34 ± 0.01	0.34 ± 0.02	0.33 ± 0.02	0.13 ± 0.07	0.25 ± 0.02	0.30 ± 0
4	5.70	1012	Ethylene glycol 2TMS isomer	tr	tr	tr	tr	tr	tr	tr	tr
8	6.66	1064	1,3 Propanediol 2TMS	0.06 ± 0.01	0.05 ± 0	0.06 ± 0	0.06 ± 0.01	0.06 ± 0	0.03 ± 0.01	0.05 ± 0	0.06 ± 0
10	6.81	1072	1,3 Propanediol 2TMS isomer	0.06 ± 0.03	0.01 ± 0.01	0.01 ± 0.01	0.03 ± 0.02	tr	tr	0.01 ± 0.01	tr
23	7.98	1137	1,2-Butanediol 2TMS isomer	0.03 ± 0.01	0.01 ± 0.01	0.01 ± 0.01	0.02 ± 0.01	0.01 ± 0	tr	0.01 ± 0	0.01 ± 0.01
30	8.59	1171	2-Octanol TMS	0.04 ± 0.01	0.02 ± 0.01	0.02 ± 0	0.03 ± 0.01	0.03 ± 0.01	0.03 ± 0.01	0.02 ± 0.01	0.03 ± 0.01
36	9.08	1198	1-Octanol TMS	0.03 ± 0.01	0.03 ± 0.01	0.03 ± 0.01	0.03 ± 0	0.03 ± 0.01	0.03 ± 0.02	0.01 ± 0.01	0.03 ± 0
43	9.91	1253	Diethylene glycol 2TMS	0.21 ± 0.03	0.15 ± 0.02	0.19 ± 0.01	0.22 ± 0.02	0.20 ± 0.02	0.07 ± 0.02	0.15 ± 0	0.18 ± 0.01
47	10.44	1288	Glycerol 3TMS	6.09 ± 2.47	0.49 ± 0.39	1.51 ± 1.94	2.41 ± 1.91	2.15 ± 0.93	0.19 ± 0.08	0.23 ± 0.06	0.27 ± 0.04
98	18.09	1886	2-Ethyl-1-dodecanol	tr	tr	tr	tr	tr	tr	tr	tr
120	20.82	2156	Octadecanol TMS	0.03 ± 0.01	0.02 ± 0.01	0.02 ± 0	0.02 ± 0	0.03 ± 0	tr	0.02 ± 0	0.02 ± 0
124	22.10	2295	Oleyl alcohol TMS	0.01 ± 0.01	0.01 ± 0	0.01 ± 0	0.02 ± 0.01	0.01 ± 0	0.01 ± 0	0.01 ± 0	0.01 ± 0
TOTAL ALCOHOLS				6.89 ± 2.51	1.05 ± 0.46	2.22 ± 1.98	3.19 ± 1.98	2.85 ± 0.96	0.51 ± 0.20	0.75 ± 0.07	0.92 ± 0.06
83	15.92	1695	1-Heptadecene	0.01 ± 0	0.01 ± 0	0.01 ± 0	0.01 ± 0.01	0.01 ± 0	0.02 ± 0.01	0.01 ± 0	0.01 ± 0
127	22.76	2369	11-Tricosene	tr	tr	tr	tr	tr	tr	tr	tr
TOTAL ALIPHATIC HYDROCARBONS				0.01 ± 0	0.01 ± 0	0.01 ± 0	0.01 ± 0.01	0.01 ± 0	0.02 ± 0.01	0.01 ± 0	0.01 ± 0.01
1	5.10	966	Alanine TMS	0.02 ± 0.01	0.02 ± 0.01	0.01 ± 0	0.02 ± 0.01	0.01 ± 0.01	0.04 ± 0.02	0.02 ± 0.02	0.02 ± 0.01
3	5.40	992	*N, N*-Dimethylglycine TMS	0.75 ± 0.53	0.09 ± 0.03	0.37 ± 0.39	0.50 ± 0.41	0.12 ± 0.01	0.37 ± 0.50	0.08 ± 0.05	0.08 ± 0.01
13	7.23	1095	Valine TMS	0.14 ± 0.08	0.04 ± 0.05	0.31 ± 0.51	0.07 ± 0.05	0.23 ± 0.11	0.01 ± 0	tr	0.01 ± 0
16	7.44	1106	Valine TMS isomer	0.02 ± 0.01	0.02 ± 0.01	0.02 ± 0.01	0.01 ± 0.01	0.02 ± 0.01	0.01 ± 0.01	0.01 ± 0	0.01 ± 0.01
17	7.54	1112	Sarcosine 2TMS	1.32 ± 1.00	0.03 ± 0.05	0.19 ± 0.32	0.60 ± 0.55	0.87 ± 0.57	0.01 ± 0	tr	tr
19	7.71	1122	Alanine 2TMS	0.01 ± 0	0.01 ± 0	0.01 ± 0	0.01 ± 0	0.02 ± 0.01	0.01 ± 0	0.01 ± 0	0.01 ± 0
21	7.82	1128	Glycine 2TMS	0.21 ± 0.28	0.01 ± 0.01	0.06 ± 0.09	0.10 ± 0.12	0.32 ± 0.23	tr	0.01 ± 0	0.01 ± 0
22	7.97	1136	Glycine 2TMS isomer	tr	0.01 ± 0	tr	0.01 ± 0	tr	0.01 ± 0	tr	tr
29	8.57	1170	Leucine TMS	0.01 ± 0	0.01 ± 0	0.01 ± 0	tr	0.01 ± 0	tr	0.01 ± 0	0.01 ± 0.01
33	8.77	1181	Isoleucine TMS	0.10 ± 0.07	0.01 ± 0	0.22 ± 0.36	0.05 ± 0.06	0.10 ± 0.08	0.01 ± 0	0.01 ± 0.01	0.01 ± 0
34	8.79	1182	Norleucine	0.08 ± 0.05	0.02 ± 0.03	0.17 ± 0.27	0.05 ± 0.04	0.12 ± 0.06	0.01 ± 0	tr	0.01 ± 0
39	9.46	1224	Valine-2TMS	0.11 ± 0.09	0.03 ± 0.04	0.08 ± 0.12	0.03 ± 0.03	0.09 ± 0.05	0.01 ± 0	0.01 ± 0	0.01 ± 0
45	10.14	1268	L-Serine 2TMS	0.22 ± 0.10	1.09 ± 0.05	0.86 ± 0.18	0.52 ± 0.44	0.30 ± 0.19	0.17 ± 0.04	0.63 ± 0.54	1.51 ± 0.23
49	10.73	1307	Threonine 2TMS	0.71 ± 0.45	0.03 ± 0.04	0.34 ± 0.56	0.15 ± 0.14	0.23 ± 0.13	0.03 ± 0.03	0.02 ± 0	0.01 ± 0.01
51	10.90	1318	Glycine 3TMS	1.05 ± 0.44	0.09 ± 0.01	1.12 ± 1.77	1.01 ± 0.87	12.82 ± 7.72	0.03 ± 0.01	0.11 ± 0.02	0.11 ± 0.01
60	11.74	1373	Serine 3TMS	0.08 ± 0.03	0.02 ± 0.02	0.03 ± 0.04	0.05 ± 0.03	0.08 ± 0.04	tr	0.01 ± 0	0.01 ± 0
61	12.12	1399	Threonine 3TMS	0.28 ± 0.08	0.04 ± 0.04	0.07 ± 0.08	0.07 ± 0.03	0.10 ± 0.06	0.05 ± 0.07	0.02 ± 0.01	0.01 ± 0
64	12.64	1438	β-Alanine 4TMS	0.41 ± 0.03	0.31 ± 0.02	0.41 ± 0.10	0.36 ± 0.01	0.40 ± 0.02	0.10 ± 0.03	0.30 ± 0.02	0.34 ± 0.01
66	13.30	1487	Aminomalonic acid 3TMS	0.01 ± 0	0.01 ± 0	0.01 ± 0.01	0.01 ± 0.01	0.01 ± 0	tr	tr	0.01 ± 0
67	13.32	1489	Aspartic acid 3TMS	0.14 ± 0.02	0.01 ± 0.01	0.03 ± 0.04	0.07 ± 0.05	0.12 ± 0.07	tr	0.01 ± 0	0.01 ± 0
70	13.94	1535	Aspartic acid 3TMS	0.09 ± 0.03	0.03 ± 0.01	0.21 ± 0.31	0.14 ± 0.12	0.12 ± 0.05	0.02 ± 0.01	0.03 ± 0.03	0.06 ± 0.02
71	13.99	1539	Pyroglutamic acid 2TMS	0.21 ± 0.08	0.07 ± 0.06	0.26 ± 0.35	0.25 ± 0.23	0.24 ± 0.08	0.01 ± 0	0.03 ± 0	0.06 ± 0.04
72	14.03	1543	3-Hydroxyproline 3TMS	0.12 ± 0.05	0.03 ± 0.02	0.29 ± 0.48	0.07 ± 0.06	0.14 ± 0.08	0.01 ± 0.01	0.01 ± 0	0.01 ± 0
76	14.47	1576	Pyroglutamic acid 2TMS isomer	6.44 ± 4.36	0.46 ± 0.10^a^*	0.93 ± 0.72	3.35 ± 2.37	0.45 ± 0.04^a^*	0.21 ± 0.09^a^*	0.39 ± 0^a^*	0.50 ± 0.07^a^*
79	15.18	1632	Glutamic acid 3TMS	0.16 ± 0.05	0.03 ± 0.04	0.24 ± 0.39	0.08 ± 0.07	0.18 ± 0.09	tr	0.01 ± 0	0.01 ± 0
80	15.31	1643	Phenylalanine 2TMS	0.07 ± 0.02	0.02 ± 0.02	0.05 ± 0.08	0.04 ± 0.02	0.03 ± 0.01	tr	tr	0.01 ± 0
99	18.28	1905	Tyrosine 2TMS	0.08 ± 0.01	0.01 ± 0.01	0.09 ± 0.14	0.05 ± 0.04	0.04 ± 0.02	tr	0.01 ± 0	0.01 ± 0.01
TOTAL AMINO ACIDS				12.85 ± 6.72	2.53 ± 0.60^b^***	6.38 ± 6.97^b^***	7.69 ± 4.44^b^***	17.16 ± 9.57	1.13 ± 0.68^b^***	1.74 ± 0.52^b^***	2.82 ± 0.33^b^***
12	7.12	1089	Caproic acid TMS	0.13 ± 0.02	0.11 ± 0.01	0.16 ± 0.01	0.15 ± 0.02	0.15 ± 0.03	0.08 ± 0.02	0.12 ± 0.01	0.14 ± 0.01
15	7.38	1103	2-Ethylhexanoic acid TMS	0.36 ± 0.07	0.27 ± 0.01	0.36 ± 0.02	0.37 ± 0.02	0.34 ± 0.03	0.16 ± 0.06	0.26 ± 0	0.32 ± 0.01
57	11.59	1364	Butyl caprylate	6.53 ± 0.54	5.54 ± 0.30	6.57 ± 0.21	6.77 ± 0.33	6.49 ± 0.17	1.89 ± 0.75	5.39 ± 0.11	5.98 ± 0.09
58	11.61	1365	Nonanoic acid, TMS	0.07 ± 0.01	0.04 ± 0	0.06 ± 0	0.07 ± 0.01	0.06 ± 0.01	0.03 ± 0.02	0.05 ± 0.01	0.06 ± 0.01
78	14.90	1609	Pimelic acid 2TMS	0.15 ± 0.01	0.14 ± 0.09	0.15 ± 0.02	0.11 ± 0.10	0.04 ± 0.01	0.06 ± 0.03	0.05 ± 0.03	0.10 ± 0.05
84	16.06	1707	Suberic acid 2TMS	0.12 ± 0.01	0.12 ± 0.01	0.14 ± 0.01	0.13 ± 0.01	0.11 ± 0.03	0.09 ± 0.01	0.09 ± 0.01	0.11 ± 0.02
90	17.20	1802	Azelaic acid 2TMS	0.29 ± 0.03	0.17 ± 0.10	0.22 ± 0.08	0.44 ± 0.39	0.13 ± 0	0.16 ± 0.08	0.11 ± 0.01	0.16 ± 0.05
96	17.72	1852	Myristic acid TMS	0.45 ± 0.09	0.23 ± 0.08	0.34 ± 0.07	0.48 ± 0.10	1.18 ± 0.57	0.11 ± 0.07	0.22 ± 0.02	0.27 ± 0.04
104	18.77	1950	Pentadecanoic acid TMS	0.21 ± 0.03	0.08 ± 0.02	0.11 ± 0.04	0.14 ± 0.03	0.12 ± 0.04	0.05 ± 0.04	0.07 ± 0	0.07 ± 0.01
109	19.59	2030	Palmitoleic acid TMS	0.65 ± 0.18	0.19 ± 0.13	0.30 ± 0.12	0.42 ± 0.13	0.60 ± 0.34	0.06 ± 0.05	0.15 ± 0.03	0.16 ± 0.04
110	19.77	2049	Palmitic acid TMS*	9.40 ± 3.18	2.22 ± 1.10^c^***	2.93 ± 1.52^c^***	5.21 ± 1.93^c^***	3.65 ± 1.26^c^***	2.00 ± 2.12^c^***	1.56 ± 0.30^c^***	1.73 ± 0.29^c^***
111	19.81	2053	Myristic acid TMS	0.02 ± 0.02	0.01 ± 0	0.01 ± 0	0.01 ± 0	0.01 ± 0	0.01 ± 0	0.01 ± 0	0.01 ± 0
113	20.37	2110	Margaric acid TMS	0.13 ± 0.04	0.04 ± 0.02	0.05 ± 0.03	0.08 ± 0.01	0.04 ± 0.02	0.02 ± 0.01	0.03 ± 0	0.04 ± 0
118	20.73	2147	Margaric acid TMS isomer	0.44 ± 0.25	0.12 ± 0.02	0.20 ± 0.08	0.22 ± 0.06	0.14 ± 0.02	0.06 ± 0.04	0.12 ± 0.01	0.13 ± 0.01
119	20.80	2154	Sarcosine ester	0.06 ± 0.05	0.02 ± 0	0.02 ± 0.01	0.03 ± 0.01	0.03 ± 0	0.01 ± 0.01	0.01 ± 0	0.02 ± 0.01
121	21.39	2216	Linoleic acid TMS	0.30 ± 0.19	0.03 ± 0.02	0.04 ± 0.03	0.08 ± 0.04	0.07 ± 0	0.02 ± 0.01	0.01 ± 0	0.03 ± 0.02
122	21.45	2223	Oleic acid TMS*	2.47 ± 1.76	0.67 ± 0.46	0.74 ± 0.07	0.58 ± 0.13	0.71 ± 0.18	0.70 ± 0.66	0.51 ± 0.23	0.56 ± 0.15
123	21.65	2244	Stearic acid TMS	5.81 ± 2.77	2.17 ± 0.44	2.79 ± 0.59	3.35 ± 0.61	2.69 ± 0.18	0.99 ± 0.81	2.07 ± 0.18	2.24 ± 0.17
125	22.41	2330	Glyceryl-glycoside TMS	0.01 ± 0	0.01 ± 0.01	0.01 ± 0	0.02 ± 0.01	0.18 ± 0.09	0.01 ± 0	0.01 ± 0	0.01 ± 0
126	22.53	2342	Nonadecanoic acid TMS	0.12 ± 0.07	0.04 ± 0	0.07 ± 0.03	0.06 ± 0.02	0.05 ± 0.03	0.04 ± 0.03	0.05 ± 0.01	0.05 ± 0.01
128	22.84	2377	Arachidonic acid TMS	1.43 ± 0.64	0.06 ± 0.03	0.11 ± 0.12	0.19 ± 0.12	0.63 ± 0.39	0.02 ± 0.01	0.02 ± 0.01	0.04 ± 0.01
129	22.91	2385	Eicosapentaenoic acid TMS	0.89 ± 0.32	0.07 ± 0.06	0.06 ± 0.07	0.33 ± 0.26	0.38 ± 0.21	0.02 ± 0	0.04 ± 0.01	0.05 ± 0.04
130	23.07	2403	1-Monomyristin 2TMS	0.26 ± 0.08	0.17 ± 0.04	0.27 ± 0.20	0.31 ± 0.10	0.25 ± 0.16	0.05 ± 0.03	0.15 ± 0.05	0.16 ± 0.06
131	23.18	2418	Eicosenoic acid TMS	0.69 ± 0.86	0.17 ± 0.03	0.22 ± 0.07	0.25 ± 0.03	0.77 ± 0.38	0.38 ± 0.35	0.18 ± 0.11	0.22 ± 0.03
132	23.38	2443	Arachidic acid TMS	0.80 ± 1.09	0.08 ± 0.02	0.14 ± 0.02	0.19 ± 0.04	0.18 ± 0.03	0.47 ± 0.45	0.13 ± 0.10	0.10 ± 0.02
133	23.78	2494	1-O-hexadecylglycerol 2TMS	0.41 ± 0.05	0.23 ± 0.06	0.34 ± 0.04	0.44 ± 0.05	0.33 ± 0.05	0.05 ± 0.02	0.21 ± 0.05	0.27 ± 0.02
134	24.36	2568	2-Monopalmitoylglycerol TMS	0.11 ± 0.07	0.04 ± 0	0.05 ± 0.01	0.06 ± 0.01	0.06 ± 0.01	0.01 ± 0.01	0.03 ± 0	0.03 ± 0
135	24.44	2578	Docosahexaenoic acid TMS	2.96 ± 1.16	0.11 ± 0.11	0.18 ± 0.23	0.28 ± 0.21	0.09 ± 0.06	0.02 ± 0.01	0.05 ± 0.01	0.10 ± 0.06
136	24.63	2603	1-Monopalmitin TMS	0.63 ± 0.27	0.28 ± 0.10	0.29 ± 0.07	0.44 ± 0.16	0.46 ± 0.15	0.06 ± 0.02	0.22 ± 0.04	0.29 ± 0.04
138	24.81	2626	Docosenoic acid TMS	3.32 ± 0.84	0.08 ± 0.03	0.18 ± 0.03	0.88 ± 1.18	0.21 ± 0.04	0.28 ± 0.22	0.13 ± 0.09	0.12 ± 0.02
140	25.84	2757	2-Monostearin TMS	0.04 ± 0.01	0.02 ± 0	0.02 ± 0.01	0.03 ± 0.02	0.02 ± 0	0.01 ± 0	0.02 ± 0	0.01 ± 0.01
141	25.96	2772	1-Monooleoylglycerol TMS	0.20 ± 0.16	0.04 ± 0.04	0.08 ± 0.05	0.11 ± 0.06	0.10 ± 0.03	0.01 ± 0	0.03 ± 0	0.03 ± 0.01
2	26.11	2791	Monostearin 2TMS isomer	0.44 ± 0.15	0.17 ± 0.02	0.21 ± 0.05	0.31 ± 0.09	0.25 ± 0.01	0.04 ± 0.03	0.19 ± 0.03	0.23 ± 0.04
144	26.32	2814	Tetracosenoic acid TMS	0.33 ± 0.16	0.14 ± 0.03	0.25 ± 0.01	0.22 ± 0.03	0.24 ± 0.03	0.09 ± 0.02	0.16 ± 0.01	0.18 ± 0.03
145	26.47	2827	Lignoceric acid TMS	0.31 ± 0.39	0.04 ± 0	0.07 ± 0.01	0.09 ± 0.01	0.06 ± 0.01	0.14 ± 0.09	0.06 ± 0.02	0.06 ± 0.01
148	27.88	2958	Hexacosanoic acid TMS	0.04 ± 0.03	0.02 ± 0	0.03 ± 0.02	0.02 ± 0	0.02 ± 0.01	0.02 ± 0.01	0.02 ± 0.01	0.01 ± 0
117	20.66	2140	3-Octadecanone	0.01 ± 0.01	0.02 ± 0.01	0.01 ± 0	0.02 ± 0.01	0.02 ± 0.01	0.02 ± 0	0.02 ± 0.01	0.01 ± 0.01
TOTAL FATTY ACIDS/ESTERS				40.61 ± 12.35^d^***	13.95 ± 3.22^c^***^,d^***	17.77 ± 3.58^c^***^,d^***	22.89 ± 5.44^c^***^,d^***	20.88 ± 3.80^c^***^,d^***	8.26 ± 6.06^c^***^,d^***	12.54 ± 1.07^c^***^,d^***	14.10 ± 1.09^c^***^,d^***
18	7.63	1117	Pipecolic acid TMS	0.08 ± 0.03	0.01 ± 0.01	0.01 ± 0.01	0.02 ± 0.01	0.01 ± 0	0.01 ± 0	0.01 ± 0.01	0.01 ± 0
24	8.12	1145	Unknown	0.20 ± 0.02	0.12 ± 0	0.17 ± 0.03	0.20 ± 0.02	0.16 ± 0.02	0.07 ± 0.02	0.12 ± 0.01	0.12 ± 0.01
26	8.43	1163	Unknown	0.13 ± 0.02	0.08 ± 0.01	0.15 ± 0.08	0.12 ± 0.01	0.13 ± 0.03	0.07 ± 0.02	0.07 ± 0.01	0.08 ± 0.01
27	8.44	1163	2-Amino-4-methylpentanamide	0.19 ± 0.07	0.09 ± 0.04	0.36 ± 0.49	0.13 ± 0.05	0.20 ± 0.08	0.03 ± 0.01	0.06 ± 0.01	0.07 ± 0.01
31	8.63	1173	Ethyl pipecolinate	0.40 ± 0.31	0.01 ± 0.01	0.08 ± 0.10	0.08 ± 0.06	0.04 ± 0.01	0.01 ± 0.01	0.01 ± 0	0.01 ± 0
40	9.59	1232	Methyl 1,2-dimethyl-5-oxo-2-pyrrolidinecarboxylate	0.02 ± 0.01	0.01 ± 0	0.01 ± 0.01	0.02 ± 0.01	0.01 ± 0	0.01 ± 0	0.01 ± 0.01	0.01 ± 0
42	9.80	1247	Urea 2TMS*	1.67 ± 0.82	2.13 ± 1.20	2.45 ± 1.68	2.33 ± 1.38	3.39 ± 0.15	0.52 ± 0.81	2.77 ± 0.11	2.53 ± 1.50
46	10.33	1280	Nicotinic acid TMS	0.03 ± 0	0.03 ± 0	0.04 ± 0.01	0.05 ± 0	0.03 ± 0	0.01 ± 0	0.03 ± 0	0.03 ± 0.01
53	11.15	1335	Picolinic acid isomer TMS	0.01 ± 0.01	0.01 ± 0	0.01 ± 0.01	0.03 ± 0.02	0.02 ± 0.02	0.01 ± 0	tr	tr
55	11.41	1352	Uracil 2TMS	0.03 ± 0.01	0.02 ± 0.02	0.03 ± 0.04	0.02 ± 0	0.04 ± 0.01	tr	0.01 ± 0	0.01 ± 0
62	12.21	1405	Cadaverine 4TMS	1.68 ± 0.16	0.56 ± 0.31	0.66 ± 0.31	1.19 ± 0.30	1.21 ± 0.23	0.22 ± 0.14	0.47 ± 0.01	0.63 ± 0.23
75	14.43	1572	Creatinine 3TMS	7 ± 5.28	0.03 ± 0.02^a^**	0.54 ± 0.87^a^*	3.56 ± 2.85	0.04 ± 0.01^a^**	0.02 ± 0^a^**	0.01 ± 0.01^a^**	0.02 ± 0.02^a^**
137	24.73	2615	Inosine 4TMS	2.15 ± 0.58	0.11 ± 0.04	0.11 ± 0.16	0.86 ± 0.72	0.07 ± 0.03	0.06 ± 0.03	0.05 ± 0	0.11 ± 0.07
143	26.22	2804	Lauryl amide	0.12 ± 0.03	0.01 ± 0.01	0.10 ± 0.01	0.09 ± 0.03	0.15 ± 0.01	0.02 ± 0	0.02 ± 0.03	0.02 ± 0.03
TOTAL NITROGENOUS COMPOUNDS				13.70 ± 7.06	3.22 ± 0.82^a^***	4.72 ± 0.40^a^***	8.69 ± 2.81	5.50 ± 0.53^a^***	1.06 ± 1.03^a^***	3.64 ± 0.07^a^***	3.67 ± 1.52^a^***
5	6.06	1031	2-Ketobutyric acid	0.01 ± 0	0.02 ± 0	0.02 ± 0.02	0.01 ± 0.01	0.03 ± 0	0.01 ± 0.01	0.02 ± 0	0.03 ± 0
6	6.48	1054	Pyruvic acid 2TMS	0.06 ± 0.02	0.04 ± 0	0.05 ± 0	0.05 ± 0.01	0.04 ± 0	0.03 ± 0.01	0.04 ± 0	0.04 ± 0
9	6.79	1071	Lactic acid 2TMS*	25.98 ± 15.45^e^***	0.55 ± 0.43^a^***^,e^***	1.77 ± 2.13^a^***^,e^***	10.01 ± 8.52^a^***	0.44 ± 0.13^a^***^,e^***	0.15 ± 0.03^a^***^,e^***	0.23 ± 0.13^a^***^,e^***	0.50 ± 0.28^a^***^,e^***
11	7.05	1085	Glycolic acid 2TMS	0.05 ± 0.01	0.05 ± 0.02	0.07 ± 0.02	0.08 ± 0.02	0.06 ± 0.01	0.02 ± 0	0.04 ± 0	0.05 ± 0.01
14	7.26	1097	Oxalic acid isomer 2TMS	0.03 ± 0.01	0.02 ± 0.01	0.03 ± 0.01	0.04 ± 0.02	0.22 ± 0.10	0.02 ± 0.01	0.02 ± 0.01	0.02 ± 0.01
20	7.72	1122	Oxalic acid 2TMS	0.01 ± 0.01	0.01 ± 0	0.01 ± 0	0.01 ± 0	0.02 ± 0.01	0.01 ± 0	0.01 ± 0	0.01 ± 0.01
25	8.25	1152	β-Lactic acid 2TMS	0.03 ± 0.02	0.03 ± 0.01	0.04 ± 0	0.04 ± 0.01	0.04 ± 0	0.02 ± 0	0.02 ± 0	0.03 ± 0
28	8.53	1168	β-Hydroxybutyric acid 2TMS	0.04 ± 0	0.02 ± 0	0.03 ± 0.02	0.05 ± 0.04	0.03 ± 0.01	0.01 ± 0.01	0.01 ± 0.01	0.02 ± 0
32	8.72	1178	Heptanoic acid TMS	tr	tr	tr	tr	tr	tr	tr	tr
35	8.83	1184	2-Ketobutyric acid TMS	0.02 ± 0.01	0.01 ± 0.01	0.02 ± 0.01	0.02 ± 0	0.03 ± 0	0.02 ± 0.01	0.01 ± 0	0.01 ± 0
37	9.31	1213	Acetoacetic acid 2TMS	0.04 ± 0.02	0.01 ± 0	0.01 ± 0	0.01 ± 0	0.04 ± 0.02	tr	0.01 ± 0	0.01 ± 0
38	9.34	1215	2-Ketobutyric acid, enol 2TMS	0.04 ± 0.01	0.01 ± 0	0.02 ± 0.01	0.02 ± 0.01	0.56 ± 0.28	0.01 ± 0.01	0.01 ± 0	0.01 ± 0
41	9.76	1243	4-Hydroxybutyric acid 2TMS	1.38 ± 0.09	1.12 ± 0.02	1.34 ± 0.03	1.42 ± 0.08	1.34 ± 0.07	0.33 ± 0.19	1.10 ± 0.05	1.31 ± 0.09
52	10.97	1323	Succinic acid 2TMS	0.41 ± 0.24	0.15 ± 0.03	0.25 ± 0.16	0.31 ± 0.13	0.19 ± 0	0.03 ± 0.01	0.13 ± 0.01	0.15 ± 0
54	11.30	1345	Glyceric acid 3TMS	0.02 ± 0.01	0.01 ± 0.01	0.01 ± 0	0.05 ± 0.04	0.01 ± 0	tr	tr	0.01 ± 0
56	11.44	1354	Fumaric acid 2TMS	0.02 ± 0.01	0.01 ± 0	0.02 ± 0.01	0.01 ± 0	0.01 ± 0	tr	0.01 ± 0	0.01 ± 0
59	11.66	1368	Maleic acid 2TMS	0.09 ± 0.07	0.05 ± 0.07	0.11 ± 0.09	0.06 ± 0.08	0.09 ± 0.04	0.01 ± 0.02	0.09 ± 0.07	0.13 ± 0
63	12.31	1413	Glutaric acid 2TMS	0.06 ± 0.01	0.05 ± 0	0.09 ± 0.03	0.08 ± 0.01	0.06 ± 0.01	0.02 ± 0.02	0.06 ± 0	0.07 ± 0.01
68	13.51	1503	Malic acid 3TMS	0.33 ± 0.09	0.03 ± 0.01	0.05 ± 0.04	0.07 ± 0.04	0.05 ± 0	0.01 ± 0	0.03 ± 0	0.03 ± 0.01
77	14.62	1586	2-Hydroxyglutaric acid 3TMS	0.01 ± 0	0.01 ± 0	0.01 ± 0.01	0.01 ± 0.01	0.02 ± 0	tr	0.01 ± 0	0.01 ± 0
94	17.59	1840	Citric acid 4TMS	0.02 ± 0	0.01 ± 0.01	0.03 ± 0.03	0.03 ± 0.02	0.04 ± 0.01	0.01 ± 0	tr	0.01 ± 0
TOTAL ORGANIC ACIDS				28.65 ± 15.84^e^***	2.21 ± 0.60^a^***^,e^***	3.96 ± 2.44^a^***^,e^***	12.38 ± 8.78^a^***	3.33 ± 0.6^a^***^,e^***	0.74 ± 0.28^a^***^,e^***	1.85 ± 0.23^a^***^,e^***	2.46 ± 0.29^a^***^,e^***
147	27.80	2951	γ-Tocopherol TMS	0.05 ± 0.05	0.01 ± 0	0.01 ± 0	0.02 ± 0	0.01 ± 0	0.01 ± 0	0.01 ± 0.01	0.01 ± 0
149	28.88	3048	Stigmastan-3,5-diene	0.30 ± 0.38	0.05 ± 0.03	0.09 ± 0.02	0.10 ± 0.01	0.07 ± 0.02	0.10 ± 0.03	0.08 ± 0.07	0.05 ± 0.01
150	29.23	3079	Cholesterol TMS	2.59 ± 0.27	0.39 ± 0.49	1.20 ± 1.35	0.71 ± 0.66	0.73 ± 0.27	0.04 ± 0.03	0.10 ± 0.02	0.29 ± 0.18
151	29.99	3145	Unknown	0.02 ± 0.02	0.01 ± 0	0.02 ± 0.01	0.02 ± 0.01	0.01 ± 0	0.01 ± 0	0.01 ± 0	0.01 ± 0
152	32.25	3343	5-Cholesten-3β-ol-7-one TMS	0.01 ± 0.01	0.01 ± 0	0.02 ± 0.02	0.02 ± 0	0.01 ± 0	0.01 ± 0	0.01 ± 0	0.01 ± 0.01
TOTAL STEROIDS/TERPENOIDS				2.98 ± 0.64	0.47 ± 0.51	1.34 ± 1.40	0.88 ± 0.66	0.82 ± 0.28	0.17 ± 0.02	0.21 ± 0.06	0.37 ± 0.19
92	17.49	1831	Arabinose 4TMS	0.07 ± 0.05	0.04 ± 0.01	0.06 ± 0.06	0.06 ± 0.02	0.12 ± 0.03	0.02 ± 0.01	0.03 ± 0	0.03 ± 0.01
93	17.57	1838	Fructofuranose 5TMS	0.03 ± 0.01	0.01 ± 0.01	0.03 ± 0.03	0.04 ± 0.02	0.07 ± 0.01	0.01 ± 0	0.01 ± 0	0.01 ± 0.01
95	17.67	1847	Sorbopyranose 5TMS	0.02 ± 0	0.02 ± 0.01	0.04 ± 0.04	0.04 ± 0.02	0.05 ± 0.01	0.01 ± 0	0.01 ± 0	0.02 ± 0.01
100	18.45	1921	Glucose 5TMS*	0.05 ± 0.02	0.03 ± 0.02	0.06 ± 0.06	0.15 ± 0.11	1.70 ± 0.78	0.01 ± 0	0.03 ± 0.01	0.05 ± 0.01
101	18.48	1924	Talopyranose 5TMS	0.04 ± 0	0.03 ± 0.01	0.04 ± 0.01	0.06 ± 0.02	0.18 ± 0.06	0.03 ± 0.02	0.03 ± 0.01	0.04 ± 0
103	18.59	1934	Mannose 5TMS	0.09 ± 0.09	0.02 ± 0.01	0.07 ± 0.06	0.14 ± 0.11	0.20 ± 0.10	0.03 ± 0.01	0.02 ± 0	0.01 ± 0
105	19.10	1981	Galactopyranose 5TMS	2.61 ± 1.39	0.06 ± 0.05	0.49 ± 0.78	0.46 ± 0.36	0.03 ± 0	0.01 ± 0	0.03 ± 0.01	0.03 ± 0.01
106	19.26	1996	Unknown sugar	0.09 ± 0.05	0.01 ± 0.01	0.02 ± 0.01	0.04 ± 0.04	0.03 ± 0.01	0.01 ± 0	0.01 ± 0	0.01 ± 0
107	19.35	2006	Glucopyranose 5TMS	0.06 ± 0.03	0.04 ± 0.03	0.09 ± 0.09	0.26 ± 0.19	2.33 ± 1.01	0.03 ± 0.01	0.05 ± 0.01	0.07 ± 0.01
116	20.62	2135	N-Acetyl-D-glucosamine 4TMS	0.10 ± 0.01	0.07 ± 0.01	0.10 ± 0.01	0.10 ± 0.01	0.10 ± 0.01	0.03 ± 0.01	0.07 ± 0	0.08 ± 0
139	25.39	2700	Sucrose 8TMS	0.12 ± 0	0.10 ± 0.12	1.10 ± 1.78	0.10 ± 0.06	2.01 ± 3.33	0.07 ± 0.04	0.10 ± 0.06	0.13 ± 0.12
146	26.65	2844	Trehalose 8TMS	0.05 ± 0.02	0.04 ± 0.01	0.20 ± 0.23	0.05 ± 0.02	0.30 ± 0.41	0.07 ± 0.03	0.03 ± 0.01	0.06 ± 0.02
TOTAL SUGARS				3.33 ± 1.47	0.47 ± 0.27	2.30 ± 3.14	1.49 ± 0.83	7.11 ± 2.31	0.31 ± 0.13	0.43 ± 0.11	0.54 ± 0.11
74	14.31	1563	L-Threonic acid 4TMS	0.09 ± 0.03	0.01 ± 0.01	0.10 ± 0.15	0.04 ± 0.03	0.05 ± 0.04	0.01 ± 0	0.01 ± 0	0.01 ± 0
91	17.24	1807	Ribonic acid 5TMS	0.30 ± 0.03	0.17 ± 0.10	0.24 ± 0.10	0.45 ± 0.39	0.85 ± 0.38	0.17 ± 0.08	0.12 ± 0.01	0.16 ± 0.05
102	18.51	1926	Gluconic acid lactone 4TMS	0.09 ± 0.01	0.07 ± 0.04	0.11 ± 0.08	0.21 ± 0.16	2.13 ± 0.86	0.10 ± 0.05	0.06 ± 0.06	0.09 ± 0.05
TOTAL SUGAR ACIDS				0.48 ± 0.05	0.25 ± 0.14	0.45 ± 0.33	0.70 ± 0.57	3.03 ± 1.28	0.27 ± 0.13	0.19 ± 0.05	0.26 ± 0.04
86	16.36	1732	Arabinitol 5TMS	0.08 ± 0.02	0.03 ± 0.04	0.03 ± 0.02	0.20 ± 0.21	0.06 ± 0.01	0.01 ± 0.01	0.03 ± 0.03	0.05 ± 0.05
97	17.81	1861	D-Pinitol 5TMS	0.34 ± 0.23	0.31 ± 0.09	0.07 ± 0.09	0.36 ± 0.15	1.20 ± 0.55	0.02 ± 0.02	0.30 ± 0.03	0.32 ± 0.05
108	19.46	2017	Myo-inositol 6TMS	0.09 ± 0.04	0.02 ± 0	0.03 ± 0.03	0.03 ± 0.02	0.10 ± 0.05	0.01 ± 0	0.01 ± 0	0.01 ± 0
112	19.89	2061	Scyllo-Inositol 6TMS	0.04 ± 0	0.03 ± 0.01	0.21 ± 0.33	0.03 ± 0.01	0.26 ± 0.11	0.02 ± 0.01	0.02 ± 0.01	0.02 ± 0.01
114	20.46	2119	Myoinositol TMS	0.75 ± 0.18	0.21 ± 0.07	0.70 ± 0.75	0.44 ± 0.07	0.61 ± 0.24	0.12 ± 0.05	0.21 ± 0.08	0.21 ± 0.05
115	20.51	2125	Myo-inositol 6TMS isomer	0.69 ± 0.20	0.21 ± 0.07	0.70 ± 0.75	0.41 ± 0.10	0.58 ± 0.26	0.14 ± 0.07	0.21 ± 0.08	0.21 ± 0.04
TOTAL SUGAR ALCOHOLS				2.00 ± 0.62	0.80 ± 0.25	1.74 ± 1.96	1.46 ± 0.18	2.81 ± 1.18	0.32 ± 0.12	0.78 ± 0.21	0.81 ± 0.03

Peak no	Rt (min)	RI	Annotation^#^	BCV	RCV	CNF	CNM	DLF	SOF	SOM	SLB
2	5.37	989	Ethylene glycol 2TMS	0.37 ± 0.02	0.33 ± 0.04	0.13 ± 0.02	0.15 ± 0.01	0.39 ± 0.03	0.35 ± 0.01	0.20 ± 0.03	0.30 ± 0.02
4	5.70	1012	Ethylene glycol 2TMS isomer	tr	tr	tr	tr	tr	tr	tr	tr
8	6.66	1064	1,3 Propanediol 2TMS	0.07 ± 0.01	0.07 ± 0	0.02 ± 0	0.03 ± 0	0.07 ± 0	0.06 ± 0.01	0.04 ± 0.01	0.07 ± 0.01
10	6.81	1072	1,3 Propanediol 2TMS isomer	0.01 ± 0	tr	tr	tr	0.01 ± 0	0.01 ± 0.01	tr	tr
23	7.98	1137	1,2-Butanediol 2TMS isomer	0.09 ± 0.02	0.05 ± 0.01	0.01 ± 0	0.01 ± 0	0.01 ± 0	0.02 ± 0	0.01 ± 0	0.01 ± 0
30	8.59	1171	2-Octanol TMS	0.04 ± 0.02	0.03 ± 0	0.03 ± 0.01	0.02 ± 0.01	0.03 ± 0.01	0.03 ± 0	0.03 ± 0.01	0.02 ± 0.01
36	9.08	1198	1-Octanol TMS	0.05 ± 0.01	0.05 ± 0	0.02 ± 0	0.02 ± 0	0.04 ± 0.01	0.06 ± 0.02	0.05 ± 0	0.03 ± 0
43	9.91	1253	Diethylene glycol 2TMS	0.31 ± 0.03	0.22 ± 0.02	0.07 ± 0.01	0.09 ± 0.01	0.22 ± 0.02	0.21 ± 0.01	0.13 ± 0.02	0.18 ± 0.01
47	10.44	1288	Glycerol 3TMS	3.46 ± 0.68	1.79 ± 0.03	0.41 ± 0.08	0.48 ± 0.12	0.81 ± 0.08	2.21 ± 1.68	1.19 ± 0.67	0.43 ± 0.09
98	18.09	1886	2-Ethyl-1-dodecanol	0.02 ± 0	0.01 ± 0	tr	tr	tr	0.01 ± 0.01	tr	tr
120	20.82	2156	Octadecanol TMS	0.06 ± 0	0.04 ± 0	0.01 ± 0	0.01 ± 0	0.03 ± 0.01	0.03 ± 0	0.01 ± 0	0.02 ± 0
124	22.10	2295	Oleyl alcohol TMS	0.01 ± 0	0.01 ± 0	0.01 ± 0	0.01 ± 0	0.01 ± 0	0.01 ± 0	0.01 ± 0	0.01 ± 0
TOTAL ALCOHOLS				4.50 ± 0.73	2.61 ± 0.02	0.72 ± 0.12	0.82 ± 0.15	1.63 ± 0.12	2.99 ± 1.70	1.68 ± 0.67	1.06 ± 0.07
83	15.92	1695	1-Heptadecene	0.01 ± 0	0.01 ± 0	0.01 ± 0	0.01 ± 0	0.01 ± 0.01	0.01 ± 0.01	0.01 ± 0.01	0.01 ± 0
127	22.76	2369	11-Tricosene	0.02 ± 0	0.01 ± 0	tr	tr	tr	0.01 ± 0.01	tr	tr
TOTAL ALIPHATIC HYDROCARBONS				0.02 ± 0	0.02 ± 0.01	0.02 ± 0	0.01 ± 0	0.01 ± 0	0.02 ± 0.01	0.01 ± 0.01	0.01 ± 0.01
1	5.10	966	Alanine TMS	0.02 ± 0.01	0.03 ± 0	0.02 ± 0.01	0.02 ± 0.01	0.02 ± 0.01	0.01 ± 0.01	0.03 ± 0.04	0.02 ± 0.01
3	5.40	992	*N, N*-Dimethylglycine TMS	0.08 ± 0.06	0.15 ± 0.05	0.25 ± 0.12	1.03 ± 0.43	0.09 ± 0.05	7.38 ± 6.45	6.54 ± 4.20	0.10 ± 0.02
13	7.23	1095	Valine TMS	0.07 ± 0.06	0.03 ± 0.01	0.16 ± 0.06	0.22 ± 0.12	0.02 ± 0	0.58 ± 0.50	0.63 ± 0.46	0.02 ± 0.02
16	7.44	1106	Valine TMS isomer	0.02 ± 0.01	0.02 ± 0.01	0.01 ± 0.01	0.01 ± 0	0.01 ± 0	0.04 ± 0.03	0.01 ± 0	0.01 ± 0.01
17	7.54	1112	Sarcosine 2TMS	0.03 ± 0.02	0.01 ± 0	0.28 ± 0.11	0.14 ± 0.07	0.01 ± 0	0.52 ± 0.47	0.85 ± 0.67	0.02 ± 0.03
19	7.71	1122	Alanine 2TMS	0.01 ± 0	0.01 ± 0	0.01 ± 0	0.01 ± 0	0.01 ± 0	0.01 ± 0.01	0.01 ± 0	0.01 ± 0.01
21	7.82	1128	Glycine 2TMS	tr	tr	0.05 ± 0.02	0.03 ± 0.02	tr	0.03 ± 0.02	0.06 ± 0.04	0.01 ± 0
22	7.97	1136	Glycine 2TMS isomer	0.01 ± 0	tr	0.01 ± 0	0.01 ± 0	tr	tr	tr	0.01 ± 0
29	8.57	1170	Leucine TMS	0.01 ± 0	0.01 ± 0	0.01 ± 0	0.01 ± 0	0.01 ± 0	0.01 ± 0.01	0.01 ± 0	0.01 ± 0
33	8.77	1181	Isoleucine TMS	0.01 ± 0	0.01 ± 0	0.06 ± 0.03	0.43 ± 0.36	0.01 ± 0	0.59 ± 0.53	1.08 ± 0.88	0.01 ± 0
34	8.79	1182	Norleucine	0.06 ± 0.04	0.02 ± 0.01	0.06 ± 0.01	0.18 ± 0.10	0.01 ± 0	0.38 ± 0.32	0.47 ± 0.34	0.01 ± 0.01
39	9.46	1224	Valine-2TMS	0.02 ± 0.01	0.01 ± 0	0.06 ± 0.02	0.08 ± 0.04	0.01 ± 0	0.15 ± 0.13	0.16 ± 0.13	0.01 ± 0.01
45	10.14	1268	L-Serine 2TMS	0.71 ± 0.48	1.41 ± 0.29	0.10 ± 0.07	0.18 ± 0.02	1.49 ± 0.06	0.80 ± 0.45	0.39 ± 0.12	1.16 ± 0.15
49	10.73	1307	Threonine 2TMS	0.09 ± 0.06	0.04 ± 0.03	0.05 ± 0.03	0.15 ± 0.08	0.03 ± 0.02	0.33 ± 0.26	0.43 ± 0.34	0.01 ± 0
51	10.90	1318	Glycine 3TMS	0.10 ± 0	0.11 ± 0.01	0.04 ± 0.01	0.08 ± 0.01	0.12 ± 0	0.24 ± 0.16	0.12 ± 0.04	0.10 ± 0.01
60	11.74	1373	Serine 3TMS	0.01 ± 0.01	0.01 ± 0	0.01 ± 0	0.02 ± 0.01	0.01 ± 0	0.02 ± 0.01	0.02 ± 0.01	0.01 ± 0
61	12.12	1399	Threonine 3TMS	0.02 ± 0.01	0.03 ± 0	0.03 ± 0	0.06 ± 0.02	0.02 ± 0	0.05 ± 0.03	0.08 ± 0.06	0.02 ± 0
64	12.64	1438	β-Alanine 4TMS	0.52 ± 0.05	0.45 ± 0.01	0.10 ± 0.02	0.16 ± 0.01	0.35 ± 0.03	0.42 ± 0.11	0.21 ± 0.03	0.34 ± 0
66	13.30	1487	Aminomalonic acid 3TMS	0.31 ± 0.08	0.20 ± 0.05	0.01 ± 0	0.01 ± 0	0.01 ± 0.01	0.01 ± 0	0.01 ± 0	0.01 ± 0
67	13.32	1489	Aspartic acid 3TMS	0.01 ± 0	0.01 ± 0	0.01 ± 0	0.01 ± 0	tr	0.05 ± 0.04	0.03 ± 0.02	0.01 ± 0.01
70	13.94	1535	Aspartic acid 3TMS	0.31 ± 0.30	0.10 ± 0.01	0.01 ± 0.01	0.03 ± 0.02	0.04 ± 0.01	0.15 ± 0.20	0.07 ± 0.04	0.03 ± 0.02
71	13.99	1539	Pyroglutamic acid 2TMS	0.48 ± 0.15	0.16 ± 0.02	0.02 ± 0.01	0.08 ± .04	0.12 ± 0.07	0.49 ± 0.39	0.32 ± 0.19	0.08 ± 0.05
72	14.03	1543	3-Hydroxyproline 3TMS	0.04 ± 0.01	0.01 ± 0.01	0.01 ± 0.01	0.04 ± 0.02	0.01 ± 0	0.56 ± 0.50	0.41 ± 0.30	0.01 ± 0.01
76	14.47	1576	Pyroglutamic acid 2TMS isomer	0.69 ± 0.07	0.73 ± 0.11	0.16 ± 0.05^a^*	0.27 ± 0.06^a^*	0.63 ± 0.08^a^*	0.55 ± 0.09^a^*	0.33 ± 0.04^a^*	0.51 ± 0.04^a^*
79	15.18	1632	Glutamic acid 3TMS	0.05 ± 0.03	0.02 ± 0	0.01 ± 0	0.03 ± 0.01	0.01 ± 0.01	0.03 ± 0.01	0.09 ± 0.06	0.01 ± 0.01
80	15.31	1643	Phenylalanine 2TMS	0.02 ± 0.01	0.01 ± 0	0.01 ± 0	0.03 ± 0.01	0.01 ± 0	0.01 ± 0	0.06 ± 0.03	0.01 ± 0.01
99	18.28	1905	Tyrosine 2TMS	0.03 ± 0.02	0.02 ± 0.01	0.01 ± 0	0.01 ± 0	0.01 ± 0	0.12 ± 0.10	0.18 ± 0.17	0.01 ± 0
TOTAL AMINO ACIDS				3.73 ± 0.58^b^***	3.60 ± 0.24^b^***	1.53 ± 0.44^b^***	3.31 ± 1.28^b^***	3.05 ± 0.26^b^***	13.53 ± 9.64	12.60 ± 7.95	2.54 ± 0.33^b^***
12	7.12	1089	Caproic acid TMS	0.19 ± 0.02	0.17 ± 0.01	0.07 ± 0.01	0.10 ± 0.01	0.18 ± 0.03	0.21 ± 0.03	0.16 ± 0.03	0.14 ± 0.02
15	7.38	1103	2-Ethylhexanoic acid TMS	0.40 ± 0.01	0.35 ± 0.03	0.15 ± 0.03	0.19 ± 0.01	0.41 ± 0.04	0.40 ± 0.02	0.25 ± 0.02	0.31 ± 0.02
57	11.59	1364	Butyl caprylate	6.99 ± 0.21	6.56 ± 0.39	1.79 ± 0.28	2.71 ± 0.29	6.93 ± 0.20	6.96 ± 0.18	4.10 ± 0.55	6.04 ± 0.06
58	11.61	1365	Nonanoic acid, TMS	0.10 ± 0.02	0.08 ± 0.02	0.03 ± 0	0.03 ± 0.01	0.06 ± 0.01	0.07 ± 0.02	0.05 ± 0.01	0.06 ± 0
78	14.90	1609	Pimelic acid 2TMS	0.17 ± 0.06	0.17 ± 0.07	0.05 ± 0.01	0.09 ± 0.02	0.18 ± 0.03	0.18 ± 0.03	0.13 ± 0.04	0.12 ± 0.05
84	16.06	1707	Suberic acid 2TMS	0.19 ± 0.04	0.22 ± 0.02	0.08 ± 0.02	0.13 ± 0.05	0.13 ± 0.01	0.20 ± 0.08	0.20 ± 0.09	0.13 ± 0.01
90	17.20	1802	Azelaic acid 2TMS	0.38 ± 0.09	0.35 ± 0.07	0.11 ± 0.01	0.16 ± 0.07	0.27 ± 0.10	0.60 ± 0.34	0.33 ± 0.12	0.23 ± 0.12
96	17.72	1852	Myristic acid TMS	1.58 ± 0.15	1.11 ± 0.05	0.20 ± 0.02	0.14 ± 0.02	0.50 ± 0.19	1.01 ± 0.44	0.49 ± 0.25	0.28 ± 0.05
104	18.77	1950	Pentadecanoic acid TMS	0.37 ± 0.01	0.25 ± 0.01	0.14 ± 0.03	0.06 ± 0	0.21 ± 0.07	0.26 ± 0.08	0.18 ± 0.10	0.12 ± 0.05
109	19.59	2030	Palmitoleic acid TMS	0.79 ± 0.09	0.49 ± 0.09	0.86 ± 0.29	0.10 ± 0.05	0.23 ± 0.06	0.29 ± 0.14	0.15 ± 0.07	0.47 ± 0.48
110	19.77	2049	Palmitic acid TMS*	13.09 ± 1.37	9.14 ± 1.12	2.67 ± 0.74^c^***	1.22 ± 0.15^c^***	4.21 ± 1.12^c^***	7.91 ± 1.47	3.07 ± 1.43^c^***	2.52 ± 1.23^c^***
111	19.81	2053	Myristic acid TMS	0.01 ± 0	0.01 ± 0	0.01 ± 0	tr	0.01 ± 0.01	0.02 ± 0.01	0.01 ± 0	0.01 ± 0
113	20.37	2110	Margaric acid TMS	0.20 ± 0.02	0.14 ± 0.03	0.12 ± 0.04	0.03 ± 0.01	0.06 ± 0.01	0.05 ± 0.01	0.03 ± 0.01	0.06 ± 0.04
118	20.73	2147	Margaric acid TMS isomer	0.42 ± 0.04	0.32 ± 0.02	0.22 ± 0.07	0.08 ± 0.02	0.24 ± 0.06	0.51 ± 0.28	0.15 ± 0.07	0.19 ± 0.08
119	20.80	2154	Sarcosine ester	0.04 ± 0.01	0.04 ± 0.01	0.01 ± 0	0.01 ± 0	0.03 ± 0.01	0.04 ± 0.01	0.02 ± 0.01	0.02 ± 0.01
121	21.39	2216	Linoleic acid TMS	0.08 ± 0.01	0.04 ± 0.01	0.04 ± 0.01	0.04 ± 0.02	0.07 ± 0.06	0.08 ± 0.06	0.04 ± 0	0.06 ± 0.06
122	21.45	2223	Oleic acid TMS*	1.34 ± 0.13	1.02 ± 0.03	1.18 ± 0.36	0.85 ± 0.51	0.95 ± 0.69	0.97 ± 0.68	0.60 ± 0.28	0.94 ± 0.61
123	21.65	2244	Stearic acid TMS	4.85 ± 0.23	3.94 ± 0.11	1.44 ± 0.46	0.99 ± 0.10	3.38 ± 0.50	4.71 ± 0.50	1.44 ± 0.26	2.48 ± 0.44
125	22.41	2330	Glyceryl-glycoside TMS	0.01 ± 0	0.01 ± 0	0.01 ± 0	0.01 ± 0	0.01 ± 0	0.04 ± 0.02	0.04 ± 0.02	0.01 ± 0.01
126	22.53	2342	Nonadecanoic acid TMS	0.09 ± 0.03	0.09 ± 0.01	0.04 ± 0	0.03 ± 0.01	0.06 ± 0.01	0.08 ± 0.03	0.03 ± 0	0.05 ± 0.03
128	22.84	2377	Arachidonic acid TMS	0.23 ± 0.03	0.14 ± 0.04	0.41 ± 0.18	0.07 ± 0.02	0.07 ± 0.02	0.25 ± 0.19	0.20 ± 0.17	0.15 ± 0.19
129	22.91	2385	Eicosapentaenoic acid TMS	0.17 ± 0.03	0.12 ± 0.02	0.35 ± 0.16	0.04 ± 0.01	0.06 ± 0.02	1.19 ± 0.99	0.53 ± 0.49	0.12 ± 0.17
130	23.07	2403	1-Monomyristin 2TMS	0.32 ± 0.04	0.36 ± 0.12	0.12 ± 0.05	0.08 ± 0.04	0.15 ± 0.04	0.14 ± 0.06	0.09 ± 0.03	0.34 ± 0.12
131	23.18	2418	Eicosenoic acid TMS	1.05 ± 0.10	0.64 ± 0.05	0.29 ± 0.08	0.20 ± 0.03	0.28 ± 0.05	1.02 ± 0.61	0.30 ± 0.09	0.25 ± 0.03
132	23.38	2443	Arachidic acid TMS	0.21 ± 0.02	0.20 ± 0.01	0.25 ± 0.03	0.15 ± 0.01	0.15 ± 0.03	0.66 ± 0.56	0.19 ± 0.04	0.13 ± 0.02
133	23.78	2494	1-O-hexadecylglycerol 2TMS	0.62 ± 0.03	0.51 ± 0.10	0.07 ± 0.02	0.10 ± 0.01	0.51 ± 0.12	0.48 ± 0.04	0.17 ± 0.03	0.32 ± 0.03
134	24.36	2568	2-Monopalmitoylglycerol TMS	0.51 ± 0.11	0.24 ± 0.04	0.02 ± 0	0.02 ± 0.02	0.08 ± 0.02	0.10 ± 0.03	0.05 ± 0.02	0.05 ± 0.02
135	24.44	2578	Docosahexaenoic acid TMS	0.21 ± 0.07	0.13 ± 0.01	0.23 ± 0.12	0.08 ± 0.01	0.12 ± 0.03	1.67 ± 1.41	0.72 ± 0.64	0.10 ± 0.10
136	24.63	2603	1-Monopalmitin TMS	2.88 ± 0.70	1.28 ± 0.15	0.13 ± 0.03	0.07 ± 0.03	0.57 ± 0.17	0.62 ± 0.23	0.20 ± 0.09	0.35 ± 0.07
138	24.81	2626	Docosenoic acid TMS	0.22 ± 0.03	0.20 ± 0.03	0.17 ± 0.02	0.13 ± 0.02	0.20 ± 0.04	0.55 ± 0.53	0.18 ± 0.03	0.14 ± 0.02
140	25.84	2757	2-Monostearin TMS	0.15 ± 0.03	0.07 ± 0.01	0.01 ± 0	0.01 ± 0	0.04 ± 0.01	0.06 ± 0.04	0.01 ± 0.01	0.02 ± 0.01
141	25.96	2772	1-Monooleoylglycerol TMS	0.60 ± 0.10	0.26 ± 0.05	0.03 ± 0	0.05 ± 0.07	0.16 ± 0.03	0.08 ± 0.02	0.03 ± 0	0.05 ± 0.03
2	26.11	2791	Monostearin 2TMS isomer	1.24 ± 0.35	0.59 ± 0.06	0.05 ± 0.01	0.05 ± 0.02	0.33 ± 0.07	0.40 ± 0.12	0.12 ± 0.04	0.24 ± 0.02
144	26.32	2814	Tetracosenoic acid TMS	0.32 ± 0.01	0.24 ± 0.02	0.07 ± 0.01	0.07 ± 0.02	0.24 ± 0.01	0.26 ± 0.06	0.11 ± 0	0.19 ± 0.01
145	26.47	2827	Lignoceric acid TMS	0.09 ± 0.01	0.08 ± 0.02	0.07 ± 0.01	0.06 ± 0.01	0.07 ± 0.01	0.21 ± 0.18	0.08 ± 0.01	0.08 ± 0.02
148	27.88	2958	Hexacosanoic acid TMS	0.02 ± 0.01	0.02 ± 0.01	0.01 ± 0	0.01 ± 0.01	0.02 ± 0.01	0.04 ± 0.01	0.02 ± 0	0.02 ± 0.01
117	20.66	2140	3-Octadecanone	0.03 ± 0.02	0.03 ± 0.01	0.01 ± 0.01	0.01 ± 0	0.02 ± 0	0.02 ± 0.02	0.01 ± 0	0.02 ± 0.01
TOTAL FATTY ACIDS/ESTERS				40.15 ± 3.61	29.61 ± 1.42^c^***	11.50 ± 2.87^c^***^,d^***	8.18 ± 1.26^c^***^,d^***	21.20 ± 2.91^c^***^,d^***	32.39 ± 3.30^c^**	14.49 ± 3.80^c^***^,d^***	16.84 ± 3.71^c^***^,d^***
18	7.63	1117	Pipecolic acid TMS	0.01 ± 0	0.01 ± 0	0.01 ± 0	0.01 ± 0.01	0.01 ± 0	0.01 ± 0.01	0.02 ± 0.01	0.01 ± 0
24	8.12	1145	Unknown	0.23 ± 0.01	0.19 ± 0.02	0.07 ± 0	0.11 ± 0.04	0.19 ± 0.02	0.25 ± 0.04	0.16 ± 0.03	0.13 ± 0
26	8.43	1163	Unknown	0.14 ± 0.03	0.14 ± 0	0.06 ± 0.01	0.10 ± 0.02	0.12 ± 0.02	0.21 ± 0.09	0.20 ± 0.10	0.09 ± 0
27	8.44	1163	2-Amino-4-methylpentanamide	0.15 ± 0.07	0.10 ± 0.01	0.09 ± 0.03	0.28 ± 0.14	0.09 ± 0.01	0.73 ± 0.57	0.88 ± 0.64	0.08 ± 0
31	8.63	1173	Ethyl pipecolinate	0.02 ± 0	0.02 ± 0	0.02 ± 0.01	0.05 ± 0.01	0.01 ± 0.01	0.36 ± 0.31	0.62 ± 0.39	0.01 ± 0
40	9.59	1232	Methyl 1,2-dimethyl-5-oxo-2-pyrrolidinecarboxylate	0.09 ± 0.08	0.04 ± 0.01	0.02 ± 0	0.01 ± 0	0.01 ± 0	0.09 ± 0.08	0.05 ± 0.03	0.02 ± 0.03
42	9.80	1247	Urea 2TMS*	0.58 ± 0.22	3.88 ± 0.32	0.40 ± 0.54	0.61 ± 0.53	2.83 ± 1.82	0.44 ± 0.22	1.40 ± 1.06	2.65 ± 1.46
46	10.33	1280	Nicotinic acid TMS	0.05 ± 0	0.05 ± 0	0.02 ± 0	0.02 ± 0	0.05 ± 0.01	0.17 ± 0.12	0.09 ± 0.05	0.04 ± 0.01
53	11.15	1335	Picolinic acid isomer TMS	0.02 ± 0.01	0.02 ± 0.01	0.01 ± 0	0.01 ± 0.01	0.01 ± 0	1.78 ± 1.60	0.76 ± 0.68	0.01 ± 0
55	11.41	1352	Uracil 2TMS	0.04 ± 0.01	0.04 ± 0	0.06 ± 0.01	0.03 ± 0.01	0.02 ± 0	0.03 ± 0.02	0.03 ± 0.01	0.01 ± 0.01
62	12.21	1405	Cadaverine 4TMS	0.88 ± 0.11	0.93 ± 0.13	0.29 ± 0.07	0.47 ± 0.09	0.66 ± 0.27	1.15 ± 0.21	0.66 ± 0.19	0.68 ± 0.36
75	14.43	1572	Creatinine 3TMS	0.04 ± 0.01^a^**	0.04 ± 0^a^**	0.02 ± 0^a^**	0.11 ± 0.17^a^**	0.04 ± 0^a^**	0.04 ± 0^a^**	0.02 ± 0.01^a^**	0.01 ± 0.01^a^**
137	24.73	2615	Inosine 4TMS	0.14 ± 0.04	0.07 ± 0.01	0.07 ± 0.06	0.07 ± 0.06	0.20 ± 0.09	0.33 ± 0.40	0.08 ± 0.06	0.02 ± 0.02
143	26.22	2804	Lauryl amide	0.20 ± 0.02	0.09 ± 0.04	0.02 ± 0.01	0.01 ± 0	0.14 ± 0.03	0.06 ± 0.02	0.03 ± 0.01	0.01 ± 0.01
TOTAL NITROGENOUS COMPOUNDS				2.58 ± 0.53^a^***	5.60 ± 0.34^a^***	1.14 ± 0.54^a^***	1.90 ± 0.36^a^***	4.35 ± 1.74^a^***	5.65 ± 2.87^a^***	4.99 ± 1.14^a^***	3.77 ± 1.04^a^***
5	6.06	1031	2-Ketobutyric acid	0.03 ± 0	0.02 ± 0	0.01 ± 0	0.02 ± 0	0.03 ± 0	0.02 ± 0.01	0.01 ± 0	0.03 ± 0
6	6.48	1054	Pyruvic acid 2TMS	0.06 ± 0.01	0.06 ± 0.01	0.02 ± 0	0.02 ± 0	0.05 ± 0.01	0.05 ± 0	0.04 ± 0.01	0.05 ± 0.01
9	6.79	1071	Lactic acid 2TMS*	1.50 ± 0.58^a^***^,e^***	0.35 ± 0.05^a^***^,e^***	0.14 ± 0.04^a^***^,e^***	0.15 ± 0.01^a^***^,e^***	0.65 ± 0.13^a^***^,e^***	1.83 ± 1.7^a^***^,e^***	1.01 ± 0.49^a^***^,e^***	0.52 ± 0.4^a^***^,e^***
11	7.05	1085	Glycolic acid 2TMS	0.17 ± 0.04	0.12 ± 0.01	0.02 ± 0	0.04 ± 0	0.07 ± 0.01	0.10 ± 0.03	0.07 ± 0.02	0.05 ± 0
14	7.26	1097	Oxalic acid isomer 2TMS	0.07 ± 0.02	0.05 ± 0.01	0.01 ± 0	0.03 ± 0	0.03 ± 0	0.06 ± 0.04	0.03 ± 0.01	0.03 ± 0.01
20	7.72	1122	Oxalic acid 2TMS	0.02 ± 0.01	0.03 ± 0.02	0.01 ± 0	tr	0.01 ± 0.01	0.01 ± 0.01	0.01 ± 0	0.01 ± 0
25	8.25	1152	β-Lactic acid 2TMS	0.07 ± 0.01	0.05 ± 0	0.03 ± 0	0.02 ± 0	0.04 ± 0.01	0.05 ± 0.01	0.03 ± 0.01	0.03 ± 0
28	8.53	1168	β-Hydroxybutyric acid 2TMS	0.03 ± 0	0.03 ± 0.01	0.01 ± 0	0.01 ± 0	0.03 ± 0.01	0.03 ± 0.01	0.02 ± 0	0.02 ± 0
32	8.72	1178	Heptanoic acid TMS	tr	tr	tr	tr	tr	tr	tr	tr
35	8.83	1184	2-Ketobutyric acid TMS	0.06 ± 0.02	0.04 ± 0	0.02 ± 0	0.01 ± 0.01	0.02 ± 0	0.02 ± 0.01	0.02 ± 0.01	0.02 ± 0
37	9.31	1213	Acetoacetic acid 2TMS	0.01 ± 0.01	0.01 ± 0	0.01 ± 0.01	0.03 ± 0.04	0.01 ± 0	0.43 ± 0.37	0.05 ± 0.06	0.01 ± 0.01
38	9.34	1215	2-Ketobutyric acid, enol 2TMS	0.02 ± 0.02	0.02 ± 0.01	0.01 ± 0	0.01 ± 0	0.02 ± 0.02	0.06 ± 0.03	0.02 ± 0.01	0.02 ± 0.01
41	9.76	1243	4-Hydroxybutyric acid 2TMS	1.48 ± 0.04	1.54 ± 0.12	0.31 ± 0.09	0.49 ± 0.06	1.51 ± 0.09	1.38 ± 0.05	0.52 ± 0.38	1.32 ± 0.11
52	10.97	1323	Succinic acid 2TMS	0.65 ± 0.10	0.44 ± 0.03	0.03 ± 0.01	0.06 ± 0.02	0.21 ± 0	0.29 ± 0.15	0.28 ± 0.15	0.17 ± 0.03
54	11.30	1345	Glyceric acid 3TMS	0.02 ± 0.01	0.01 ± 0	tr	0.01 ± 0	0.01 ± 0.01	0.02 ± 0	0.01 ± 0	0.01 ± 0
56	11.44	1354	Fumaric acid 2TMS	0.23 ± 0.07	0.15 ± 0.03	tr	tr	0.02 ± 0	tr	0.01 ± 0	0.01 ± 0
59	11.66	1368	Maleic acid 2TMS	0.06 ± 0.08	0.01 ± 0	0.01 ± 0.01	0.02 ± 0.01	0.06 ± 0.09	0.13 ± 0.10	0.05 ± 0.02	0.05 ± 0.07
63	12.31	1413	Glutaric acid 2TMS	0.06 ± 0	0.06 ± 0	0.02 ± 0.01	0.04 ± 0	0.08 ± 0.02	0.09 ± 0.03	0.06 ± 0	0.08 ± 0.02
68	13.51	1503	Malic acid 3TMS	0.42 ± 0.34	0.15 ± 0.02	0.01 ± 0	0.02 ± 0	0.03 ± 0.01	0.03 ± 0.01	0.07 ± 0.04	0.03 ± 0.01
77	14.62	1586	2-Hydroxyglutaric acid 3TMS	0.02 ± 0	0.01 ± 0	tr	tr	0.01 ± 0	0.02 ± 0.01	0.01 ± 0.01	0.01 ± 0.01
94	17.59	1840	Citric acid 4TMS	0.69 ± 0.21	0.23 ± 0.05	0.01 ± 0	0.02 ± 0.02	0.02 ± 0	0.26 ± 0.21	0.05 ± 0.03	0.01 ± 0.01
TOTAL ORGANIC ACIDS				5.67 ± 1.06^a^***^,e^**	3.36 ± 0.04^a^***^,e^***	0.71 ± 0.08^a^***^,e^***	1.02 ± 0.13^a^***^,e^***	2.90 ± 0.06^a^***^,e^***	4.87 ± 2.42^a^***^,e^**	2.35 ± 1.19^a^***^,e^***	2.48 ± 0.43^a^***^,e^***
147	27.80	2951	γ-Tocopherol TMS	0.01 ± 0	0.01 ± 0	0.01 ± 0.01	0.01 ± 0	0.02 ± 0	0.04 ± 0.04	0.01 ± 0	0.01 ± 0
149	28.88	3048	Stigmastan-3,5-diene	0.26 ± 0.06	0.24 ± 0.06	0.11 ± 0.06	0.08 ± 0.03	0.10 ± 0.01	0.23 ± 0.17	0.12 ± 0.03	0.11 ± 0.05
150	29.23	3079	Cholesterol TMS	4.93 ± 0.80	2.16 ± 0.35	1.97 ± 0.71	0.29 ± 0.01	0.93 ± 0.35	5.86 ± 4.82	1.90 ± 1.56	1.12 ± 1.63
151	29.99	3145	Unknown	0.74 ± 0.22	0.49 ± 0.16	0.01 ± 0	0.01 ± 0	0.03 ± 0.01	0.03 ± 0	0.01 ± 0.01	0.02 ± 0.01
152	32.25	3343	5-Cholesten-3β-ol-7-one TMS	0.51 ± 0.12	0.30 ± 0.08	0.01 ± 0	0.01 ± 0	0.03 ± 0.01	0.02 ± 0.01	0.02 ± 0	0.01 ± 0
TOTAL STEROIDS/TERPENOIDS				6.44 ± 1.06	3.21 ± 0.45	2.11 ± 0.76	0.40 ± 0.01	1.11 ± 0.35	6.18 ± 4.61	2.06 ± 1.60	1.27 ± 1.69
92	17.49	1831	Arabinose 4TMS	0.21 ± 0.08	0.10 ± 0.02	0.04 ± 0	0.08 ± 0.03	0.05 ± 0	0.85 ± 0.70	0.57 ± 0.40	0.04 ± 0.02
93	17.57	1838	Fructofuranose 5TMS	0.24 ± 0.07	0.09 ± 0.01	0.01 ± 0	0.03 ± 0.02	0.03 ± 0.01	0.04 ± 0.01	0.08 ± 0.06	0.02 ± 0.01
95	17.67	1847	Sorbopyranose 5TMS	0.08 ± 0.02	0.04 ± 0	0.01 ± 0.01	0.03 ± 0.01	0.04 ± 0.01	0.04 ± 0.02	0.14 ± 0.08	0.02 ± 0.02
100	18.45	1921	Glucose 5TMS*	0.31 ± 0.04	0.17 ± 0.05	0.01 ± 0	0.04 ± 0.02	0.05 ± 0.01	0.48 ± 0.37	1.32 ± 1.04	0.03 ± 0.01
101	18.48	1924	Talopyranose 5TMS	0.08 ± 0.01	0.05 ± 0.01	0.02 ± 0	0.03 ± 0.01	0.05 ± 0.01	0.08 ± 0.01	0.13 ± 0.09	0.04 ± 0.01
103	18.59	1934	Mannose 5TMS	0.28 ± 0.02	0.15 ± 0.02	0.03 ± 0.01	0.04 ± 0.01	0.04 ± 0.03	0.61 ± 0.48	0.35 ± 0.24	0.05 ± 0.03
105	19.10	1981	Galactopyranose 5TMS	0.18 ± 0.04	0.28 ± 0.20	0.02 ± 0	0.03 ± 0.01	0.10 ± 0.04	0.20 ± 0.21	0.04 ± 0.02	0.04 ± 0.03
106	19.26	1996	Unknown sugar	0.09 ± 0.04	0.04 ± 0.01	0.02 ± 0	0.02 ± 0.01	0.02 ± 0.01	0.13 ± 0.10	0.07 ± 0.05	0.01 ± 0
107	19.35	2006	Glucopyranose 5TMS	0.46 ± 0.09	0.24 ± 0.09	0.05 ± 0.02	0.08 ± 0.04	0.07 ± 0.03	0.82 ± 0.63	2.33 ± 1.82	0.05 ± 0.01
116	20.62	2135	N-Acetyl-D-glucosamine 4TMS	0.16 ± 0.01	0.13 ± 0	0.02 ± 0	0.04 ± 0.01	0.11 ± 0.01	0.11 ± 0.01	0.11 ± 0.05	0.09 ± 0
139	25.39	2700	Sucrose 8TMS	0.32 ± 0.16	0.11 ± 0.04	0.05 ± 0	0.21 ± 0.15	0.12 ± 0.03	0.09 ± 0.03	0.06 ± 0.03	0.25 ± 0.20
146	26.65	2844	Trehalose 8TMS	0.11 ± 0.05	0.07 ± 0.05	0.03 ± 0.02	0.04 ± 0.02	0.06 ± 0.02	0.04 ± 0.02	0.03 ± 0.01	0.04 ± 0.03
TOTAL SUGARS				2.53 ± 0.57	1.47 ± 0.07	0.33 ± 0.05	0.66 ± 0.27	0.73 ± 0.10	3.50 ± 2.49	5.22 ± 3.87	0.68 ± 0.30
74	14.31	1563	L-Threonic acid 4TMS	0.03 ± 0.01	0.01 ± 0	0.03 ± 0.01	0.08 ± 0.04	0.01 ± 0	0.22 ± 0.19	0.45 ± 0.32	0.01 ± 0
91	17.24	1807	Ribonic acid 5TMS	0.38 ± 0.14	0.11 ± 0.01	0.12 ± 0.01	0.17 ± 0.06	0.28 ± 0.10	0.23 ± 0.07	0.24 ± 0.22	0.24 ± 0.12
102	18.51	1926	Gluconic acid lactone 4TMS	0.50 ± 0.08	0.32 ± 0.07	0.12 ± 0.02	0.10 ± 0.03	0.09 ± 0.01	0.55 ± 0.39	1.75 ± 1.31	0.07 ± 0.02
TOTAL SUGAR ACIDS				0.91 ± 0.21	0.44 ± 0.07	0.26 ± 0.01	0.34 ± 0.11	0.38 ± 0.10	1.00 ± 0.52	2.44 ± 1.55	0.31 ± 0.11
86	16.36	1732	Arabinitol 5TMS	0.21 ± 0.09	0.11 ± 0.11	0.02 ± 0	0.01 ± 0.01	0.09 ± 0.11	0.14 ± 0.08	0.07 ± 0.02	0.08 ± 0.06
97	17.81	1861	D-Pinitol 5TMS	1.62 ± 0.49	0.77 ± 0.05	0.02 ± 0.01	0.03 ± 0.03	0.41 ± 0.37	0.08 ± 0.03	0.06 ± 0.04	0.35 ± 0.11
108	19.46	2017	Myo-inositol 6TMS	0.04 ± 0.02	0.03 ± 0	0.01 ± 0	0.01 ± 0.01	0.01 ± 0	0.05 ± 0.03	0.03 ± 0.02	0.01 ± 0
112	19.89	2061	Scyllo-Inositol 6TMS	0.05 ± 0.01	0.04 ± 0.01	0.01 ± 0.01	0.02 ± 0	0.03 ± 0	0.10 ± 0.07	0.32 ± 0.24	0.03 ± 0.02
114	20.46	2119	Myoinositol TMS	0.84 ± 0.14	0.64 ± 0.07	0.13 ± 0.01	0.04 ± 0.01	0.25 ± 0.08	0.63 ± 0.46	0.64 ± 0.57	0.42 ± 0.12
115	20.51	2125	Myo-inositol 6TMS isomer	0.80 ± 0.09	0.64 ± 0.08	0.16 ± 0.05	0.14 ± 0.04	0.19 ± 0.04	0.62 ± 0.40	0.68 ± 0.52	0.42 ± 0.12
TOTAL SUGAR ALCOHOLS				3.57 ± 0.83	2.22 ± 0.28	0.35 ± 0.05	0.26 ± 0.08	0.99 ± 0.38	1.63 ± 1.05	1.81 ± 1.40	1.31 ± 0.34

^#^All metabolites showed a matching score above 800, tr: traces (< 0.01 mg/g), *Annotations confirmed with standards, ^a^significantly different from SAM-2, ^b^significantly different from TGF, ^c^significantly different from BCV, ^d^significantly different from SOF, ^e^significantly different from SAF-1, *p < 0.05, **p < 0.01, ***p < 0.001, Two-way analysis of variance (ANOVA) followed by Tukey’s Post-Hoc test was performed.

#### Fatty acids/esters

Lipid profile is one of the key indexes of fish roe quality being the main nutrient and energy source among other macronutrients^7^. Consequently, fatty acids/esters accounted for the major metabolite class in all examined caviar and roe samples (Table 2), notably in male *Sparus aurata* roe (SAM-2) and black caviar (BCV) (approximately 40 mg/g, *p* < 0.001), followed by female *Sepia officinalis* roe (SOF) (32.4 mg/g, *p* < 0.001 compared to other samples except RCV). Likewise, saturated fatty acids were detected at the highest levels in BCV and SAM-2 being rich in palmitic acid (peak 110) at 13.1 and 9.4 mg/g, and stearic acid (peak 123) at 4.9 and 5.8 mg/g, respectively. Palmitic acid plays a major role in lipid metabolism and energy production, serving as a precursor of palmitoyl-coenzyme A^22^. However, it should be noted that several dietary guidelines have focused on reducing saturated fatty acid intake to decrease the risk of obesity-related disorders^23^.

Esters of saturated fatty acids i.e. butyl caprylate (peak 57) found at the highest level in BCV and SOF at 6.9 mg/g, likely to impart antimicrobial properties in these roe types. Butyl caprylate is incorporated in food, pharmaceuticals, and cosmetics owing to its distinctive fruity flavor^24^.

With regards to monounsaturated fatty acids, similarly SAM-2 and BCV were most rich in oleic (peak 122) (1–2 mg/g), eicosenoic (peak 131) (0.7–1 mg/g) and docosenoic acids (peak 138) (3.3 and 0.2 mg/g), with the latter reported to possess antioxidant and anti-inflammatory effects^25^.

Among omega-3 PUFAs in roe samples, eicosapentaenoic acid (peak 129) and docosahexaenoic acid (peak 135) were the most abundant in SAM-2 (0.9 and 2.9 mg/g) and SOF (1.19 and 1.7 mg/g, respectively).

#### Alcohols

Glycerol (peak 47) represented the major alcohol in all caviar and roe samples, with SAM-2 found richest at 6.1 mg/g followed by BCV and SAF-1 (3.5 and 2.4 mg/g, respectively). In addition to fish roes as a source of glycerol, it is reported that glycerol is added to the packaged sturgeon caviar to impart a glossy look and prevent them from adhering^26^.

#### Organic acids

Organic acids represented the second major metabolite class in caviar and roe samples, specifically SAM-2 (28.7 mg/g, *p* < 0.001) and SAF-1 samples (12.4 mg/g, *p* < 0.001 compared to other samples except BCV and SOF where *p* < 0.01), represented by lactic acid as major form at 26 and 10 mg/g. The enrichment of lactic acid is consistent with previous reports on other fish roe such as *Oncorhynchus keta* (salmon), *Cyclopterus lumpus* (lumpfish), *Mallosus villosus* (capelin), *Oncorynchus mykiss* (rainbow trout), *Esox lucius* (pike), *Gadus morhua* (cod), Alaska *Theragra chalcogramma* (pollock)^10^. Lactic acid exerts potential preservative action in raw roe, functioning as an acidulant to lower pH, limit microbial growth, and enhance storage stability, especially in roe types such as pike and Alaska pollock^10^.

Most roe samples demonstrated low levels of 4-hydroxybutyric acid, from 0.31 mg/g in roe of female crab *Charybdis natator* (CNF) up to 1.5 mg/g in red caviar (RCV). This neuromodulator has a sedative effect, acting as a GABA precursor^27^.

#### Amino acids

Amino acids were most rich in female collector urchin *Tripneustes gratilla* roe; TGF at 17.2 mg/g (*p* < 0.001), especially enriched in glycine (peak 51, 12.8 mg/g) in agreement with Schmidt et al.^7^, followed by SOF and SAM-2 at 13.5 and 12.9 mg/g, respectively. Meanwhile, SOF contained high levels of *N, N*-dimethylglycine (peak 3) at 7.4 mg/g.

*N, N*-dimethylglycine is reported to exert an immunostimulant effect and to improve physical and mental performance^28^. Other essential amino acids such as valine, leucine, isoleucine, threonine, and phenylalanine were especially abundant in common cuttlefish samples i.e., SOF and SOM. These amino acids are of nutritional value in roe being critical for protein synthesis^29^ and to improve performance, health, and immunity^30^.

High content of pyroglutamic acid (represented by peaks 71 and 76) was detected in roe of male and female *S. aurata* (SAM-2 and SAF-1) at 6.65, 3.6 mg/g, respectively, likely produced via enzymatic and non-enzymatic reactions mediating its formation. Pyroglutamic acid contributes to the sensory properties of processed foods, being able to sequester the bitter taste of amino acids such as valine, leucine, and isoleucine, and to elicit the more favored umami taste. Moreover, pyroglutamic acid possesses antimicrobial, antidiabetic and anti-hyperlipidemic properties^31^, suggesting that *S. aurata* (SAM-2 and SAF-1) presents a good source of that key amino acid with the best umami taste among examined fish roe.

The conditionally essential amino acid glycine (peaks 21, 22, and 51), along with its biosynthetic precursors sarcosine (peak 17) and L-serine (peak 45) were among the major identified amino acids in the examined caviar and roe samples (Table 2). Glycine plays a vital role in the biosynthesis of porphyrins (such as heme) and glutathione, cytoprotection, and neurotransmission^32^. Additionally, β-Alanine (peak 64) was enriched in most samples. This amino acid is of nutraceutical interest being used to improve muscular performance^33^.

#### Nitrogenous compounds

Likewise in the case of some amino acids accumulation, *S. aurata* (SAM-2 and SAF-1) samples were found most rich in nitrogenous compounds (13.7 and 8.7 mg/g, respectively) with creatinine (peak 75) as major form in both samples (4–7 mg/g). Next, remarkable levels of urea (peak 42) were detected in all caviar/roe samples (0.4–3.9 mg/g) with red caviar (RCV) being the richest. The anti-nutrient nitrogenous compound cadaverine (peak 62) was found at 1.68 mg/g in SAM-2, which is a biogenic amine produced as a decomposition product resulting from the bacterial decarboxylation of lysine. Hence, its presence is suggestive for fermentation reaction that might have occurred in these samples. The high levels of urea and creatinine are suggestive to more active protein metabolism indicating a higher nutritional quality^34^.

#### Sugars/sugar alcohols

The collector urchin sample (TGF) and the common cuttlefish sample (SOM) were found richest in total sugars (7.1 and 5.2 mg/g, respectively), with β-D-glucopyranose (peak 107) as the main sugar. Sugars remain the least studied class in caviar/roe, with higher carbohydrate content in sea urchin than cuttlefish^7^. A moderate sugar level was observed in black caviar (BCV) (2.5 mg/g), slightly higher than red caviar (RCV) (1.5 mg/g).

Other detected sugars included β-D-galactose (2.61 mg/g in SAM-2) and sucrose (2.01 mg/g in TGF). With regard to sugar alcohols, myo-inositol was the major form detected at higher levels in black caviar (BCV) (1.7 mg/g) and *S. aurata* roe (SAM-2) (1.5 mg/g), though at considerably lower levels compared to free sugars.

#### Steroids/terpenoids

Steroids/terpenoids, secondary metabolite class, were found more prominent in BCV and SOF at 6 mg/g, represented by cholesterol (peak 150) as the most abundant form at 5 mg/g. Fatty fish, e.g., salmon, is known to accumulate high cholesterol levels in roe^7^. On the other hand, except for ridged swimming crab roe (CNF), the other crustacean samples (PPF-1, PPF-2, and CNM) showed less cholesterol levels (0.1, 0.3, and 0.3 mg/g, respectively) than fish roes in line with^7^.

### Multivariate data analysis of caviar and roe samples GC–MS datasets

To provide insight into caviar metabolome heterogeneity concerning interspecies and gender-related variability, different models of multivariate data analysis were implemented in an unsupervised i.e., PCA and HCA, and a supervised method, i.e., OPLS-DA.

Relative similarities and variabilities among different roe samples were first unveiled by the unsupervised multivariate analysis tool PCA. This model covered 57% of the total variance embedded in samples. Although some replicates of *Sparus aurata* segregated in the right lower quadrant of the PCA score plot (Supplementary Fig. S3), no other discrimination or grouping of samples could be achieved. Additionally, this model had unsatisfactory prediction power.

**Fig. 2:**
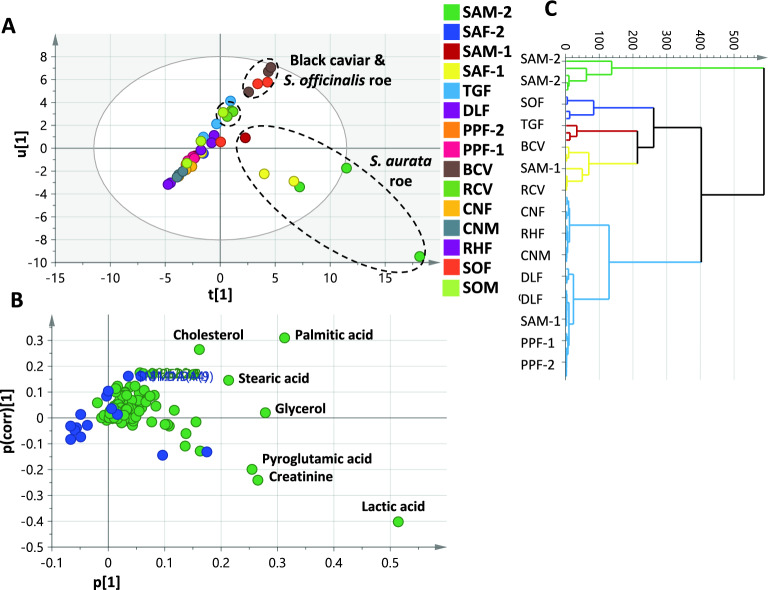
GC–MS-based orthogonal partial least squares discriminant analysis (OPLS-DA) of caviar/roe metabolome explaining 56% of the total variance (n = 3). (**A**) OPLS-DA score plot. (**B**) OPLS-DA loading plot with contributing metabolites assigned. (**C**) OPLS-DA derived dendrogram. For samples codes, refer to Table 1.

To improve predictability and discrimination among samples, OPLS-DA model was constructed using all roe samples from different families to obtain an initial comprehensive outline of compositional differences among samples, with the model accounting for 56% of the total variance. The OPLS-DA score plot (Fig. 2A) did not provide a clear discrimination among most of the samples except for those obtained from *S. aurata* represented by 4 samples: SAM-1, SAF-1, SAM-2, and SAF-2. Indeed, three of the 4 *S. aurata* roe samples segregated towards the right side of the score plot with positive PC1 values (SAM-2, SAF-1, and SAM-1), whereas two specimens (SAM-2 and SAF-1) clustered uniquely negative to PC2. Additionally, black caviar (BCV) and cuttlefish *Sepia officinalis* roe (SOF) as well as the red caviar sample (RCV) clustered in the right upper quadrant of the score plot (Fig. 2A). Several metabolites from different classes contributed towards the segregation of *S. aurata* samples, as revealed from loading plot (Fig. 2B), including pyroglutamic acid, creatinine and lactic acid. The highest content of pyroglutamic acid, creatinine and lactic acid was detected in *S. aurata*, namely SAM-2 (*p* < 0.05, 0.01, and 0.001, respectively) and SAF-1 roes and accounting for their segregation along PC2 (Fig. 2B). Interestingly, SAM-2 and SAF-1 were the most enriched in nitrogenous compounds and organic acids. It is worth mentioning that palmitic acid may also mediate for the clustering of BCV, SOF, and RCV, positively contributing to PC1 and PC2 (Fig. 2B). Substantial level of palmitic acid was found in these samples with BCV being the richest (*p* < 0.001 compared to all other samples except SOF, RCV, and SAM-2) and in agreement with quantification results at 13.1 mg/g (Table 2). Other lipids contribute to such clustering, including stearic acid and cholesterol, with the latter being most abundant in the SOF sample (5.9 mg/g). Interestingly, *S. officinalis* roe (SOF) represents a good fat source considering its high content of omega-3 PUFA (i.e., eicosapentaenoic acid and docosahexaenoic acid) alongside γ-tocopherol, in agreement with previous studies^12^ with a favored n-3/n-6 ratio of 8.6. Meanwhile, the collector urchin roe sample (TGF) presented the least n-3/n-6 ratio of 0.7, and following previously reported data of sea urchin roe^35^.

The OPLS-DA-derived dendrogram (Fig. 2C) placed SAM-2 distinctly in a separate cluster, indicating its unique composition, whereas BCV, RCV, and SOF were nested in a branch of the other cluster. Aside from that, the model did not succeed in providing good discrimination of the remaining samples. Separate models were thus created for caviar samples from each family to achieve better segregation, higher prediction power and to improve markers identification for each taxon.

#### Metabolic variability in *Sparus aurata* and *Rhabdosargus haffara* roe, Sparidae Family

**Fig. 3:**
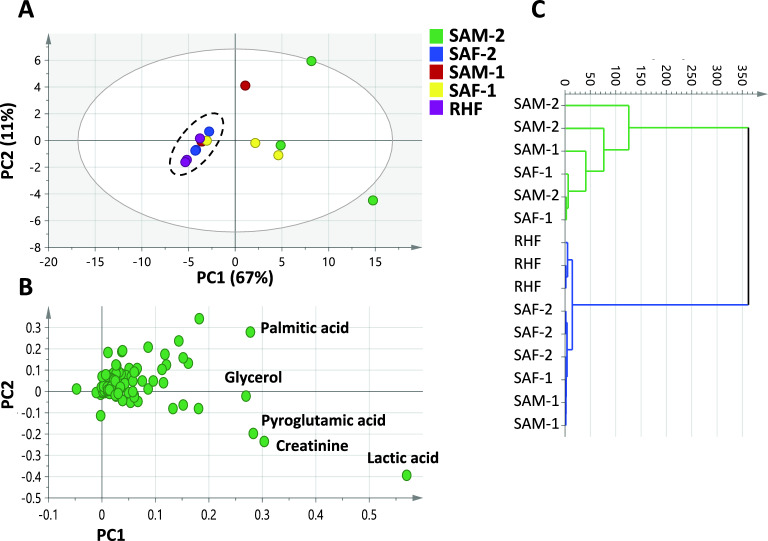
GC–MS-based principal component analysis (PCA) and hierarchical cluster analysis (HCA) of Sparidae roe metabolome (n = 3). (**A**) PCA score plot of PC1 versus PC2 describing 67% and 11% of the total variance, respectively. (**B**) Loading plot with the most contributing metabolites assigned. (**C**) HCA derived dendrogram. For samples codes, refer to Table 1.

A separate PCA model was generated for roe samples belonging to the family Sparidae. Sparidae, commonly known as porgies or seabreams, includes many popular food fish of remarkable commercial importance e.g., *S. aurata* (gilt-head bream)^36^. The rapid development in gilt-head bream production in the Mediterranean Sea led to the saturation of its market emphasizing the need to develop unconventional value-added products^37^. Sparidae is represented by one *R. haffara* roe sample from the Red Sea; RHF and 4 *S. aurata* roe samples from the Mediterranean Sea*,* namely SAM-1, SAF-1, SAM-2, and SAF-2 (Table 1). The first two Principal Components (PC-1 and PC-2) explained 78% of the total variance among Sparidae. The model revealed clustering of *R. haffara* sample (RHF) alongside the female *S. aurata* sample (SAF-2) with negative score values along PC1, versus the positioning of *S. aurata* SAM-2 and SAF-1 roe on the other side with positive PC1 values (Fig. 3A). Such clustering pattern was attributed for the rich content of palmitic acid, glycerol, pyroglutamic acid, creatinine and lactic acid in SAM-2 and SAF-1 among all Sparidae accessions (Fig. 3B). In fact, SAM-2 and SAF-1 showed the highest content of amino acids, fatty acids, and organic acids in Sparidae (Table 2). Moreover, enriched levels of nitrogenous compounds represented by creatinine and cadaverine were observed in SAM-2 and SAF-1 among all examined caviar/roe samples, not only Sparidae. Although cadaverine level is still within the acceptable limit, the intake of this biogenic amine should be carefully controlled for consumers’ safety, as it can potentiate the toxic effects of other biogenic amines, leading to nervous and cardiovascular adverse effects^38,39^, and warranting for its monitoring in plant and animal-based food products. Similarly, the HCA dendrogram grouped SAM-2 and SAF-1, while RHF and SAF-2 were positioned in a separate cluster (Fig. 3C). Both RHF and SAF-2 samples displayed the least contents of amino acids, fatty acids, organic acids, sugars, and nitrogenous compounds among Sparidae, suggestive of a less nutritive value. No clear discrimination was observed between *R. haffara* (RHF) and *S. aurata* (SAF-2) despite being of different taxa suggesting close metabolite composition in the subfamily Sparinae^40^.

#### Metabolome classification of crab roe (*Portunus pelagicus* and *Charybdis natator*, Portunidae Family) in context to taxa and gender type

**Fig. 4:**
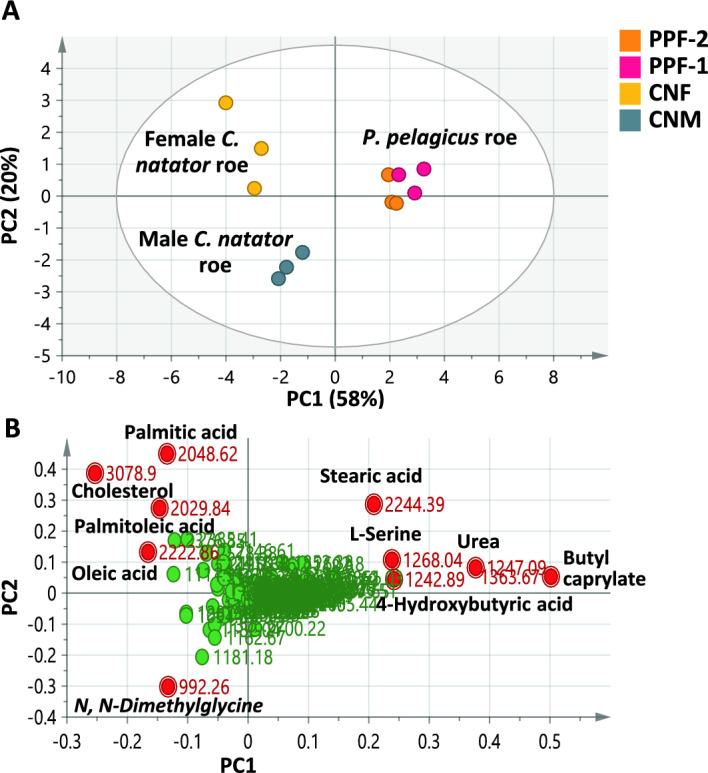
GC–MS-based PCA of Portunidae roe metabolome (n = 3). (**A**) PCA score plot of PC1 versus PC2 describing 58% and 20% of the total variance, respectively. (**B**) Loading plot with the contributing metabolites assigned. For samples codes, refer to Table 1.

PCA modeling of crab roe samples belonging to family Portunidae represented by *P. pelagicus* (PPF-1 and PPF-2) from the Mediterranean Sea and *C. natator* (CNM and CNF) from the Red Sea (Table 1), accounting for 78% of the total variance, clearly separated crab roe of *P. pelagicus* (PPF-1 and PPF-2) from *C. natator* (CNM and CNF) alongside PC1 (Fig. 4A). This finding was in agreement with the previously reported DNA barcoding-based phylogeny that placed *P. pelagicus* distant from *C. natator*^41^. Interestingly, male and female *C. natator* roe specimens (CNM and CNF, respectively) were segregated along PC2, revealing gender-based differences in Portunidae samples, which have yet to be confirmed from other taxa for results to be conclusive.

Metabolites accounting for such segregation along PC1 (Fig. 4B) included butyl caprylate, urea, serine, 4-hydroxybutyric acid, and stearic acid, found more abundant in *P. pelagicus* samples (PPF-1 and PPF-2) among the 4 examined Portunidae samples. On the other hand, oxylipids represented by palmitic, oleic, and palmitoleic acids alongside cholesterol segregated female *C. natator* (CNF) in the left upper quadrant of the score plot. Specifically, CNF was the richest Portunidae sample in these fatty metabolites. In contrast, *N, N*-dimethylglycine accounted for the separation of male *C. natator* (CNM). Indeed, male specimens were the richest in amino acids among all examined Portunidae samples, versus the richness of females in lipids. These findings match the gender-related lipid variation in fish and crab meat and gonads, where females exhibited significantly higher lipid content than males. Female crabs are known to accumulate more fat reserve to provision the developing eggs and in some Asian countries, female crabs are highly prized due to their unique aroma and texture^42–44^. However, more samples are required to strengthen this conclusion.

#### Gender variability within *Charybdis natator*, *Sparus aurata*, and *Sepia officinalis* roes

Considering the identification of a gender effect on roe composition in *C. natator* (Fig. 4), it was of interest to identify variation in male and female fish roe within other taxa for results to be conclusive using supervised OPLS-DA. A series of supervised OPLS-DA models were constructed. In the first model (Supplementary Fig. S4), male *C. natator* roe samples (CNM) were modeled against female (CNF) to identify markers for each gender type. As expected, and in line with the PCA model (Fig. 4), amino acids e.g., *N*, *N*-dimethylglycine, and isoleucine appeared enriched in male *C. natator* roe, compared with cholesterol, palmitic acid, and palmitoleic acid found more abundant in female crab roe (OPLS-DA score plot, Fig. S4). It should be noted that differences between male and female *C. natator* roe were, though not statistically significant, with a *p* value of 0.326 necessitating larger-scale study.

Two other OPLS-DA models were generated to classify male (SAM-1 & SAM-2) and female (SAF-1 & SAF-2) *S. aurata* samples, and between male (SOM) and female (SOF) *S. officinalis* samples. These models, though, showed weak prediction power with high permutation *p* values suggesting no significant differences between male and female roe of these taxa.

#### Metabolome classification of black caviar vs red caviar

**Fig. 5:**
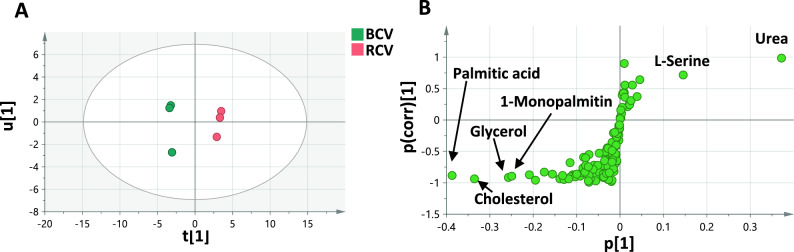
(**A**) OPLS-DA score plot derived from modelling black caviar (BCV) against red caviar (RCV) explaining 87% of the total variance (n = 3). (**B**) S-plot shows the covariance p[1] against the correlation p(corr) [1] of the discriminating component variables. Selected assigned variables are highlighted in the S-plot (validation metrics provided in Fig. S5 and VIP scores in Fig. S6 and Table S1).

Genuine black caviar, the most valued edible roe product, is obtained from sturgeon fish (*Huso* and *Acipenser* spp.), whereas its related inferior belongs to acipenseriformes species *Polyodon spathula*^1^. Fish roe obtained from non-sturgeon origin is used as a caviar substitute, such as salmon roe (commonly known as red caviar)^2^. To assess interspecies variability between black (BCV) and red caviar (RCV) and markers for each taxon, BCV was modeled against RCV in a separate OPLS-DA model. The model showed R2 and Q2 values of 0.99 indicating a good classification performance and validated using permutation test with 20 times and CV-ANOVA which demonstrated that the model is not over fitted (Supplementary Fig. S5). As illustrated in the OPLS-DA score plot (Fig. 5A), BCV and RCV were segregated along PC1 which represents 71% of the total variance. These markers contributing to such segregation are highlighted in the OPLS-DA-derived S-plot (Fig. 5B). The fatty metabolites, namely palmitic acid, cholesterol, and 1-monopalmitin, as well as glycerol, were responsible for the segregation of black caviar negative to PC1. The VIP scores of the most discriminatory variables are shown in Supplementary Fig. S6 and Table S1. In contrast, red caviar segregation to the right side of the score plot was attributed to its richness in urea (3.9 mg/g) and L-serine (1.4 mg/g), indicating active protein metabolism. In terms of total amino acid content in red and black caviar, similar levels were detected at 3.6 and 3.7 mg/g, respectively, and in compliance with previous reports^13,45^. Meanwhile, quantification results of black caviar revealed its richness in palmitic acid (13.1 mg/g) and 1-monopalmitin (2.9 mg/g), and the second richest in cholesterol (4.9 mg/g) and glycerol (3.5 mg/g) among all roe samples. Interestingly, both black and red caviar were found rich in nutrients, especially fatty acids/esters, which can be related to their ecological habitat. Sturgeons and salmon are anadromous fish that spawn in freshwater poor in nourishment and hence lay eggs rich in nutrients to ensure adequate supply to the new generation^10,13^. Black caviar recorded superior amounts of fatty acids/esters (40.2 mg/g) than red caviar (29.6 mg/g) (*p* < 0.001) in agreement with Mol et al.^45^. In contrast, Bledsoe et al. reported a different profile with higher crude lipid content in salmon caviar than black caviar, concomitant with substantial levels of cholesterol in salmonoid roe^2^. Comparative profiling of crude lipid content in black and red caviar from different origins is required using specific lipid extraction methods for results to be more conclusive.

#### Classification of genuine black caviar from non-sturgeon roe substitutes

**Fig. 6:**
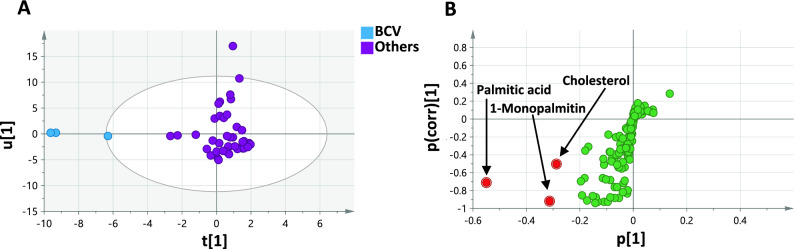
(**A**) OPLS-DA score plot derived from modelling black caviar (BCV) against other roe samples describing 47% of the total variance (n = 3). (**B**) S-plot shows the covariance p[1] against the correlation p(corr) [1] of the discriminating component variables. Contributing markers are highlighted (validation metrics provided in Fig. S7 and VIP scores in Fig. S8 and Table S2).

Considering the high value of black caviar, it was of interest to use models to discriminate between genuine black caviar obtained from sturgeons and other roe samples of non-sturgeon origin. BCV was modeled against other roe samples to visualize differences and identify markers unique to BCV from other common roe products. The model showed a good fitness and predictive power as implied by R2 and Q2 (0.78 and 0.67) and validated using permutation test with 20 times and CV-ANOVA (Supplementary Fig. S7). The model successfully discriminated BCV as an outlier from the remaining samples as illustrated in the OPLS-DA score plot (Fig. 6A), reflecting a quite divergent chemical composition. Variables that most influenced such segregation included palmitic acid (*p* < 0.001 compared to all non-sturgeon roe, except SOF, RCV, and SAM-2), 1-monopalmitin ester, and cholesterol as highlighted in the S-plot (Fig. 6B) in compliance with the previous model (Fig. 5) with their VIP scores depicted in Supplementary Fig. S8 and Table S2.

In all models employed to differentiate between various caviar/roe types, lipid profile, especially the saturated fatty acid palmitic acid and cholesterol, played a major role in discrimination, posing them as discriminatory markers in QC of caviar/roe products^1,45,46,47^. Variability in fatty acids among different caviar/roe samples was mainly quantitative rather than qualitative, in agreement with earlier reports^45^. With regards to gender influence, no significant differences were found between male and female roe samples in the examined taxa with no gender-specific metabolite markers.

However, the findings of the present study should be considered in light of some limitations. The relatively small sample size may undermine the statistical power and restrict generalizability. Future research will require large-scale studies with larger sample sizes spanning several collection sites to strengthen statistical comparisons and place the study in a broader context. Meanwhile, environmental influences of water temperature, salinity, and diet can compromise the metabolomic data by introducing variables which cannot be controlled^17^. Larger standardized studies are warranted to validate and extend the present findings.

## Conclusion

The study provided detailed metabolite profiling of different caviar and roe samples obtained from 10 commercially important taxa representing both male and female fish, and aquatic animal specimens, highlighting their functional potential and metabolic heterogeneity. A total of 139 metabolites were identified and quantified, including fatty acids/esters, alcohols, amino acids, organic acids, sugars, nitrogenous compounds, and steroids/terpenoids. The most prevalent metabolite class among examined caviar and roe samples was fatty acids/esters. The relative high levels of omega-3 polyunsaturated fatty acids exemplified by eicosapentaenoic acid and docosahexaenoic acid detected in roe of male gilt-head bream *Sparus aurata* and female common cuttlefish *Sepia officinalis* suggest their potential relevance for nutritional or functional food applications. Moreover, *S. officinalis* roe presented rich γ-tocopherol content, with best n-3/n-6 ratio up to 8.6, posing it as good fat source among all examined caviar and roe samples. Another metabolite class detected in caviar and roe samples was alcohol likely derived from fat hydrolysis. Amino acids, critical for protein synthesis, were abundant in the collector urchin *Tripneustes gratilla* exemplified by glycine and *N, N*-dimethylglycine as major forms. The rich content of pyroglutamic acid in gilt-head bream roe may contribute to its characteristic sensory attributes, though this requires confirmation through sensory analysis. Interspecies and gender-related variability was further explored via multivariate modeling of GC–MS dataset, where lipids especially palmitic acid/ester and cholesterol, were the main contributors in the discrimination of caviar from non-sturgeon roe samples. Such extensive metabolite profiling accentuates heterogeneity in chemical profile between various caviar and roe types facilitating the quality control, authenticity assessment, and nutritional labeling of caviar and roe products. This study opens the door for further investigations on potential health benefits of caviar and fish roe as functional ingredients and targeted breeding programs to improve selected traits such as omega-3 content and umami taste.

## Supplementary Information

Supplementary Information 1

Supplementary Information 2

## Data Availability

Data including raw metabolomics files will be made available upon request. Contact M.A. Farag at [mohamed.farag@pharma.cu.edu.eg] or N. Ibrahim at [nehal.sabry@pharma.asu.edu.eg].
