# Supplementary material for: GC–MS-based metabolome classification of sturgeon caviar and fish roe samples reveals unique caviar signatures, interspecies and gender variabilities
Source: Sci Rep. 2026 Feb 17;16:7195. doi: 10.1038/s41598-026-36474-6 (PMC12920915; doi:10.1038/s41598-026-36474-6)
Supplement: Supplementary file 1 — Supplementary Information 1 [file 41598_2026_36474_MOESM1_ESM.docx]

**GC-MS-based metabolome classification of sturgeon caviar and fish roe samples reveals unique caviar signatures, interspecies and gender variabilities**

**Nehal Ibrahim^a*^, Amira R. Khattab^b,c^, Ashraf S. Mohammad^d^, Montasser A. Al-Hammady^d^, Iriny Ayoub^a^, Mohamed A. Farag^e^****^*^**

^a^Pharmacognosy Department, Faculty of Pharmacy, Ain Shams University, Cairo, 11566, Egypt.

^b^Pharmacognosy Department, College of Pharmacy, Arab Academy for Science, Technology and Maritime Transport, Alexandria 1029, Egypt.

^c^Graduate School in Alamein, Arab Academy for Science, Technology & Maritime Transport, Alamein, Egypt.

^d^National Institute of Oceanography and Fisheries (NIOF), Cairo 11516, Egypt.

^e^Pharmacognosy Department, College of Pharmacy, Cairo University, Cairo, Egypt, Kasr El Aini St., P.B. 11562.

^*^Corresponding author: Mohamed A. Farag, Pharmacognosy Department, College of Pharmacy, Cairo University, Cairo, Egypt, Kasr El Aini St., P.B. 11562. E-mail address: [mohamed.farag@pharma.cu.edu.eg](mailto:mohamed.farag@pharma.cu.edu.eg) and Nehal Ibrahim, Pharmacognosy Department, Faculty of Pharmacy, Ain Shams University, Cairo, 11566, Egypt. E-mail address: [nehal.sabry@pharma.asu.edu.eg](mailto:nehal.sabry@pharma.asu.edu.eg)

**Table S1.** Retention time (RT), retention index (RI), major ions, matching score, annotation level, VIP scores, Fold changes (Log2FC) and p-values of the most discriminatory variables in OPLS-DA modeling of black caviar (BCV) against red caviar (RCV) samples based on GC-MS derived dataset.

| **Variable RT (min)** | **Variable RI** | **Annotation****^#^** | **Matching Score** | **Class** | **Annotation Level** | **VIP Score*** | **log2FC** | **p-value** |
| --- | --- | --- | --- | --- | --- | --- | --- | --- |
| 19.77 | 2048.62 | Palmitic acid TMS  *m/z* 117, 73, 75, 313, 132, 129 | 943 | Fatty acid/ester | Level 1 | 2.98 | -1.67 | 2.34e-02 |
| 9.80 | 1247.09 | Urea 2TMS  *m/z* 147, 189, 73, 148, 45, 74 | 939 | Nitrogenous compound | Level 1 | 1.89 | 1.23 | 3.87e-02 |
| 29.23 | 3078.9 | Cholesterol TMS  *m/z* 129, 368, 73, 329, 43, 57 | 933 | Steroid/  terpenoid | Level 2 | 2.12 | -1.45 | 1.85e-02 |
| 10.44 | 1287.77 | Glycerol 3TMS  *m/z* 73, 147, 103, 205, 117, 75 | 952 | Alcohol | Level 2 | 3.45 | -1.92 | 1.03e-02 |
| 24.63 | 2602.8 | 1-Monopalmitin TMS  *m/z* 371, 73, 43, 147, 372, 57 | 907 | Fatty acid/ester | Level 2 | 1.45 | -0.56 | 4.12e-02 |
| 10.14 | 1268.04 | L-Serine 2TMS  *m/z* 73, 116, 132, 75, 45, 57 | 923 | Amino acid | Level 2 | 1.76 | 0.87 | 5.67e-02 |

**^#^** All metabolites showed a matching score above 800.

*VIP > 1.0 indicates statistically significant contribution to sample discrimination.

**Table S2.** Retention time (RT), retention index (RI), major ions, matching score, annotation level, VIP scores, Fold changes (Log2FC) and p-values of the most discriminatory variables in OPLS-DA modeling of black caviar (BCV) against other roe samples based on GC-MS derived dataset.

| **Variable RT (min)** | **Variable RI** | **Annotation^#^** | **Matching Score** | **Class** | **Annotation Level** | **VIP Score*** | **log2FC** | **p-value** |
| --- | --- | --- | --- | --- | --- | --- | --- | --- |
| 19.77 | 2048.62 | Palmitic acid TMS  *m/z* 117, 73, 75, 313, 132, 129 | 943 | Fatty acid/ester | Level 1 | 1.974 | -0.685 | 2.14e-01​ |
| 29.23 | 3078.9 | Cholesterol TMS  *m/z* 129, 368, 73, 329, 43, 57 | 933 | Steroid/  terpenoid | Level 2 | 0.978 | -1.170 | 9.02e-02​ |
| 24.63 | 2602.8 | 1-Monopalmitin TMS  *m/z* 371, 73, 43, 147, 372, 57 | 907 | Fatty acid/ester | Level 2 | 0.991 | 0.022 | 9.72e-01​ |
| 10.44 | 1287.77 | Glycerol 3TMS  *m/z* 73, 147, 103, 205, 117, 75 | 952 | Alcohol | Level 2 | 2.342 | -1.830 | 2.14e-02​ |
| 9.80 | 1247.09 | Urea 2TMS  *m/z* 147, 189, 73, 148, 45, 74 | 939 | Nitrogenous compound | Level 1 | 1.562 | 0.466 | 2.52e-01​ |
| 10.14 | 1268.04 | L-Serine 2TMS  *m/z* 73, 116, 132, 75, 45, 57 | 923 | Amino acid | Level 2 | 1.986 | 0.295 | 6.12e-01​ |

**^#^** All metabolites showed a matching score above 800.

*VIP > 1.0 indicates statistically significant contribution to sample discrimination.

.


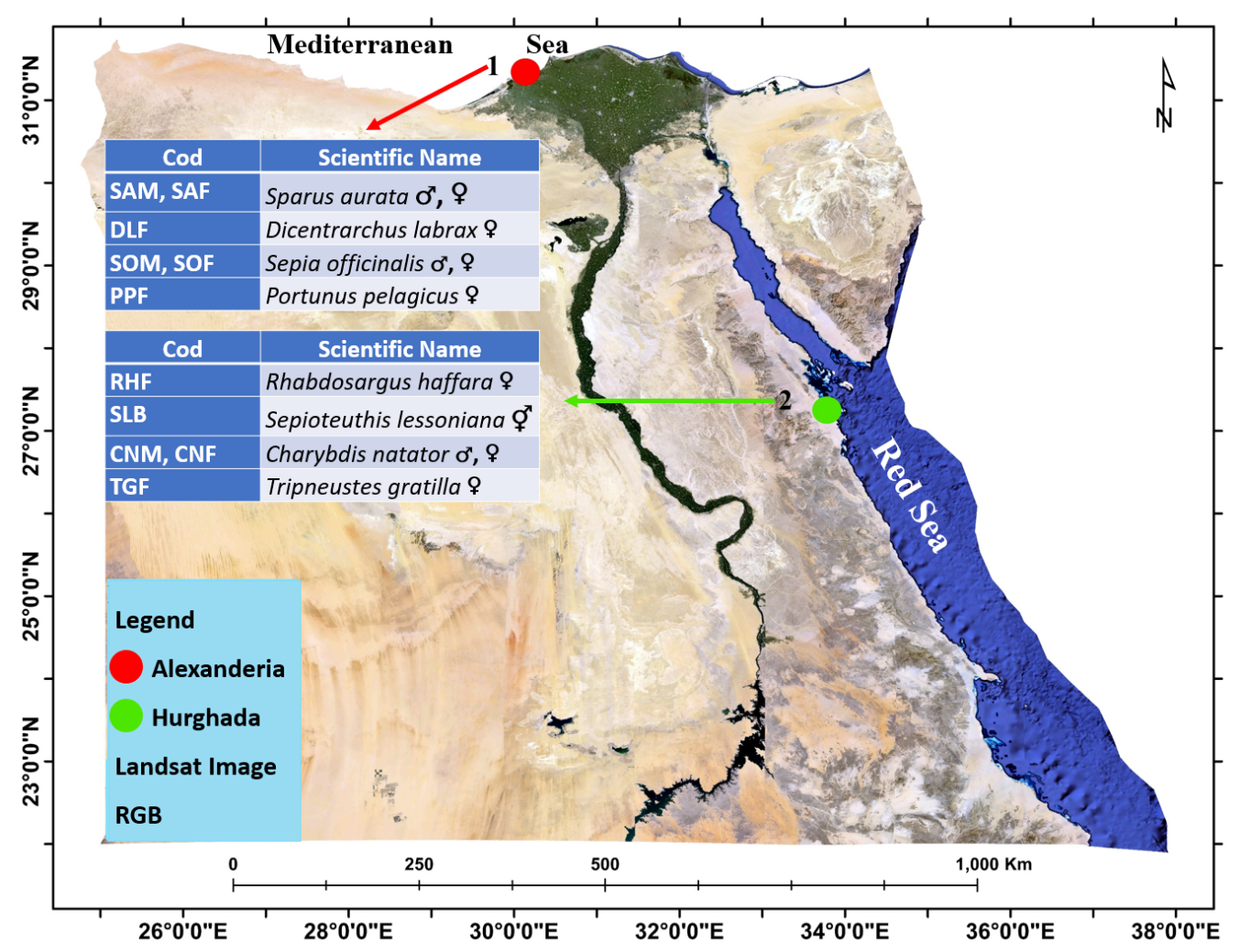


**Code**

**Code**

**Figure S1.** Sampling locations on the Mediterranean Sea in Alexandria and Red Sea in Hurghada. Map lines delineate study areas and do not necessarily depict accepted national boundaries.

**GC/MS response**


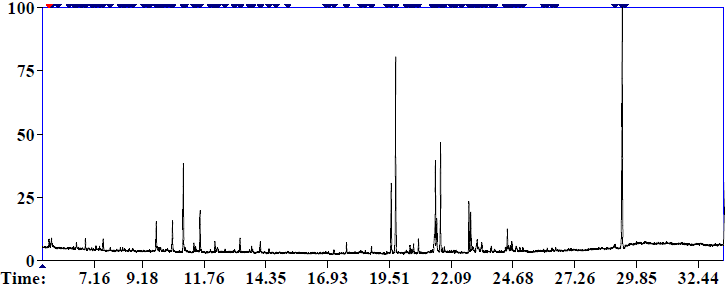


**150**

**110**

**109**

**122**

**123**

**128**

**135**

**41**

**47**

**9**

**17**

**62**

**96**

**104**

**113**

**118**

**CNF**

**GC/MS response**

**GC/MS response**

KFR ii


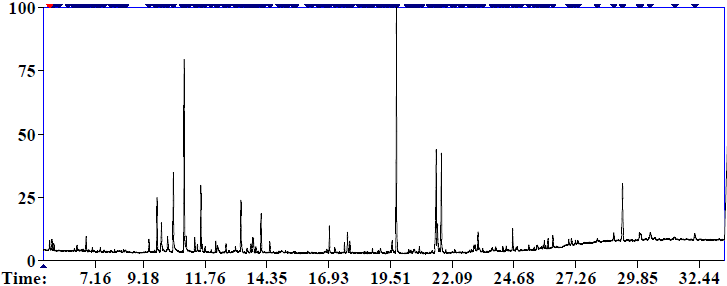


**47**

**41**

**57**

**110**

**123**

**122**

**150**

**96**

**136**

**131**

**109**

**9**

**RCV**


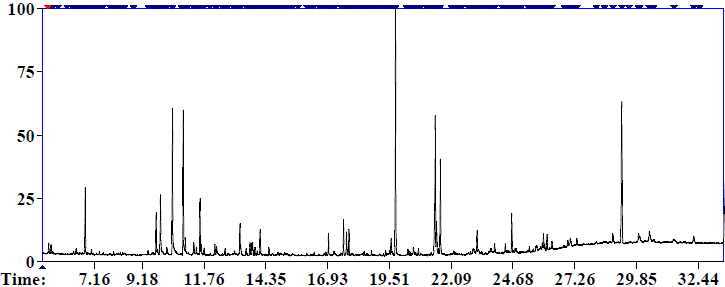


**110**

**150**

**122**

**123**

**136**

**9**

**47**

**57**

**41**

**94**

**97**

**131**

**BCV**


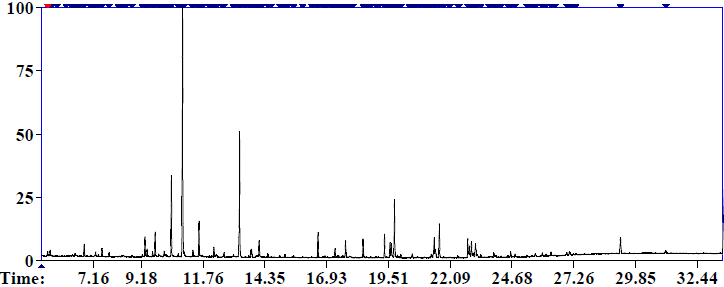


**51**

**47**

**57**

**110**

**123**

**122**

**150**

**9**

**38**

**41**

**91**

**96**

**100**

**107**

**128**

**131**

**TGF**

**GC/MS response**


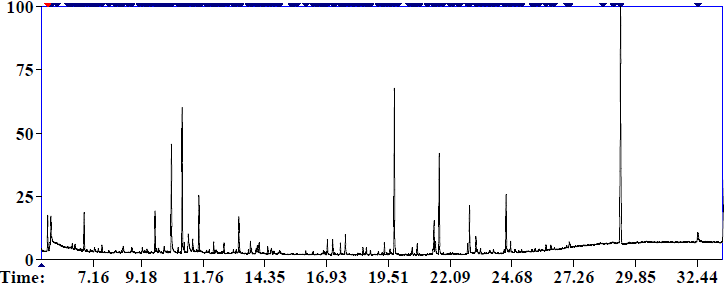


**150**

**110**

**123**

**122**

**129**

**135**

**131**

**9**

**41**

**47**

**53**

**57**

**3**

**96**

**62**

**90**

**92**

**64**

**66**

**SOF**

**GC/MS response**

**GC/MS response**


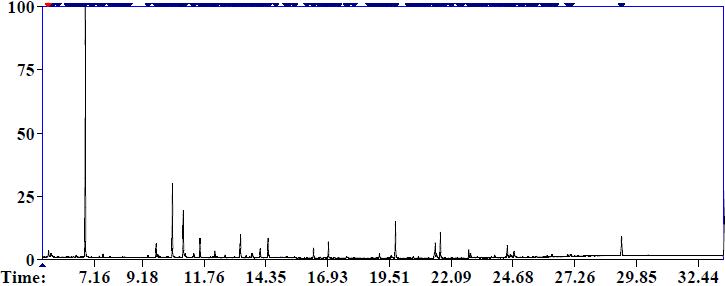


**150**

**110**

**122**

**123**

**9**

**41**

**57**

**47**

**51**

**62**

**66**

**75**

**128**

**135**

**137**

**SAM-2**

**Retention time (min)**

**Figure S2.** GC-MS chromatograms of selected caviar/roe specimens as analyzed post-silylation. Samples codes are explained in Table 1. Peaks numbers of annotated metabolites follow those listed in Table 2.

**Retention time (min)**

**A**

**B**

**SAM-2**

**SAF-2**

**SAM-1**

**SAF-1**

**TGF**

**DLF**

**PPF-2**

**PPF-1**

**BCV**

**RCV**

**CNF**

**CNM**

**RHF**

**SOF**

**SOM**

SAM-2

SAM-2

SOF

BCV

RCV

SAM-1

TGF

SOF

SOM

CNF

CNM

DLF

SAM-1

PPF-2

PPF-2

**C**

**PC2 (19%)**

**PC1 (38%)**

**Pyroglutamic acid**

**Creatinine**

**Lactic acid**

**Palmitic acid**

**Stearic acid**

**Glycerol**

**PC2**

**PC1**

**Figure S3.** GC-MS based principal component analysis (PCA) and hierarchical cluster analysis (HCA) of all caviar and roe samples (n = 3). **(A)** PCA score plot of PC1 versus PC2 describing 38% and 19% of the total variance, respectively. **(B)** Loading plot with the most contributing metabolites assigned. **(C)** HCA derived dendrogram. For samples codes, refer to **Table 1**.

**u[1]**

**t[1]**

**CNF**

**CNM**

**A**

**Cholesterol**

**Palmitic acid**

**Palmitoleic acid**

**Butyl caprylate**

***N, N*-Dimethylglycine**

**Isoleucine**

**B**

**Figure S4. (A)** OPLS-DA score plot derived from modeling male (CNM) against female *Charybdis natator* (CNF) roe samples (n = 3). **(B)** S-plot shows the covariance p[1] against the correlation p(cor)[1] of the discriminating component variables. Selected assigned variables are highlighted in the S-plot. The OPLS-DA model reflects trends rather than significant differences (*p* value = 0.326).

**
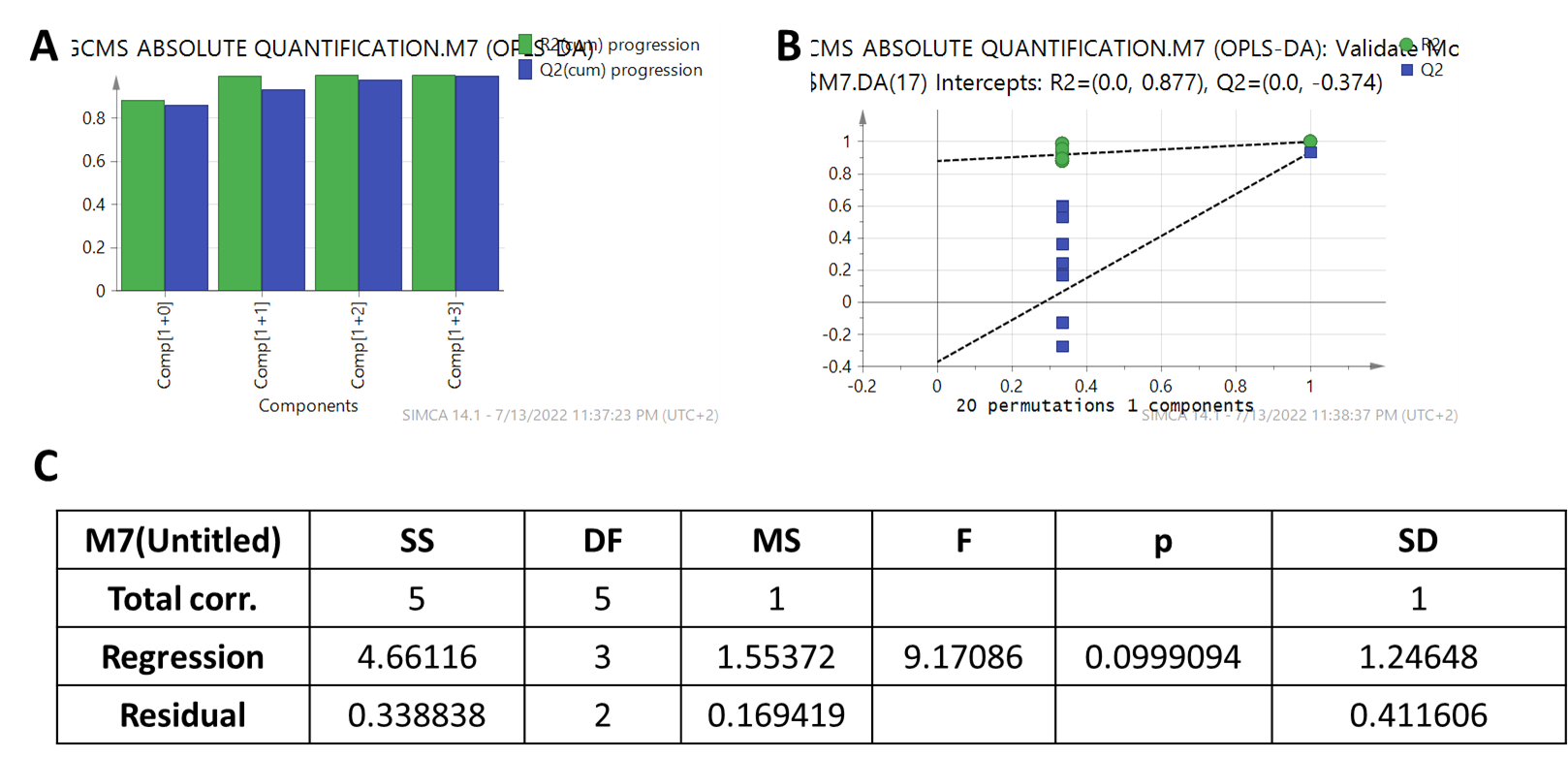
**

**Figure S5.** OPLS-DA model optimization and validation parameters for modeling black caviar (BCV) against red caviar (RCV) samples based on GC-MS derived dataset. **(A)** The diagnostic metrics R2Y and Q2 as function of number of principal components. **(B)** Permutation test, n = 20. **(C)** CV-ANOVA to assess for model statistical significance.

**Figure S6.** Variable Importance in Projection (VIP) scores for the most discriminatory variables in modeling black caviar (BCV) against red caviar (RCV) samples.


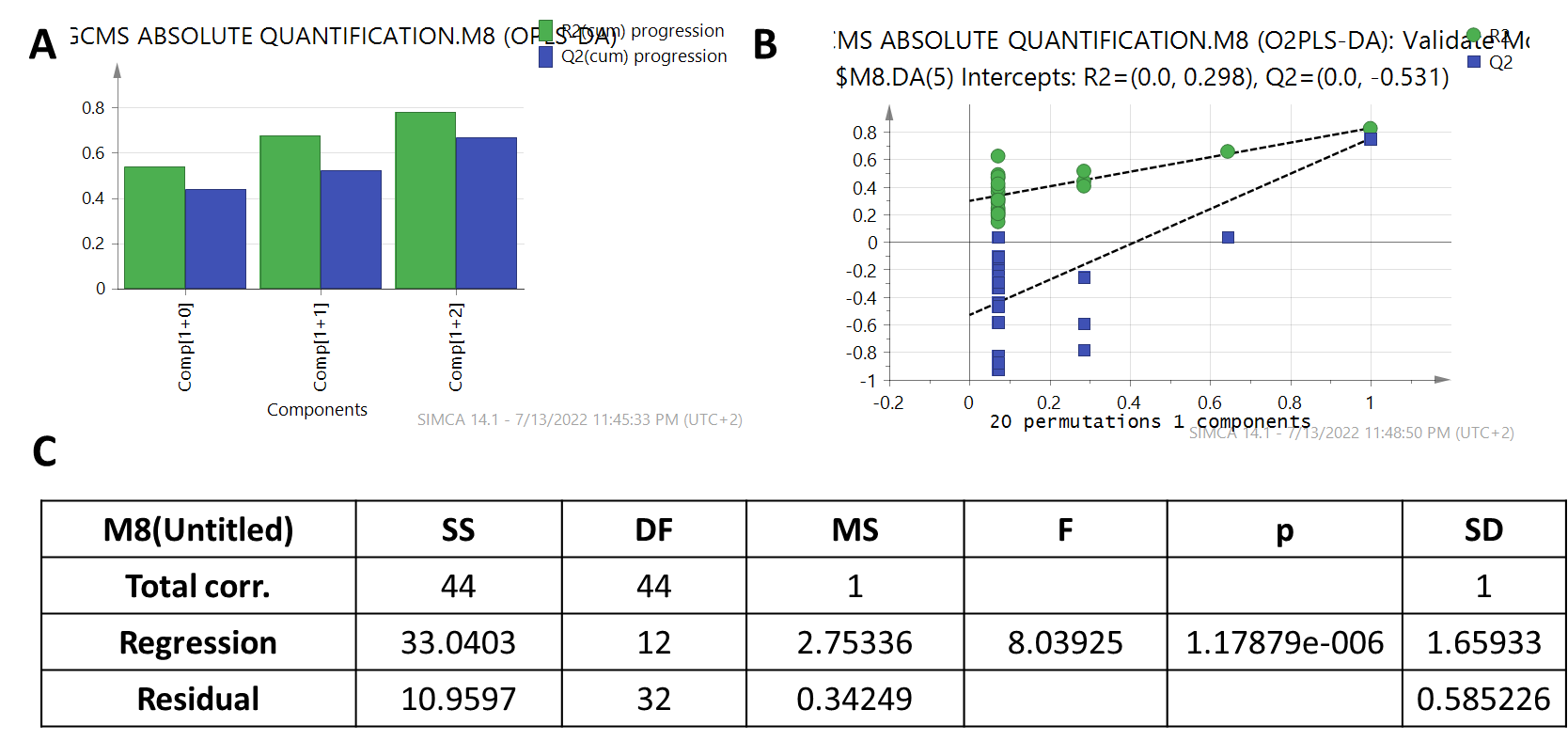


**Figure S7.** OPLS-DA model optimization and validation parameters for modeling black caviar (BCV) against other roe samples based on GC-MS derived dataset. **(A)** The diagnostic metrics R2Y and Q2 as function of number of principal components. **(B)** Permutation test, n = 20. **(C)** CV-ANOVA to assess for model statistical significance.

**Figure S8.** Variable Importance in Projection (VIP) scores for the most discriminatory variables in modeling black caviar (BCV) against other roe samples.
